# Geographic variation in the determinants of ectoparasite faunas’ species richness: fleas and gamasid mites parasitic on small mammals from 6 biogeographic realms

**DOI:** 10.1017/S0031182025100371

**Published:** 2025-06

**Authors:** Boris R. Krasnov, Vasily I. Grabovsky, Natalia Korallo-Vinarskaya, Maxim V. Vinarski, Angel Luis Robles Fernandez, Irina Khokhlova

**Affiliations:** 1 Mitrani Department of Desert Ecology, Swiss Institute for Dryland Environmental and Energy Research, Jacob Blaustein Institutes for Desert Research, Ben-Gurion University of the Negev, Sede Boqer Campus, Midreshet Ben-Gurion, Israel; 2 French Associares Institute for agriculture and Biotechnology of Drylands, Jacob Blaustein Institutes for Desert Research, Ben-Gurion University of the Negev, Sede Boqer Campus, Midreshet Ben-Gurion, Israel; 3 Laboratory for the Study of Parasitic Arthropods, Zoological Institute of the Russian Academy of Sciences, Saint-Petersburg, Russian Federation; 4 Laboratory of Macroecology and Biogeography of Invertebrates, Saint-Petersburg State University, Saint-Petersburg, Russian Federation; 5 Kansas Biological Survey & Center for Ecological Research, The University of Kansas, Lawrence, KS 66047 USA

**Keywords:** biogeographic realms, fleas, mammals, mites, parasite sharing, species richness

## Abstract

We investigated the effects of body mass, geographic range size, the within-range richness of host assemblages (diversity field) and the habitat breadth of small mammalian hosts from 6 biogeographic realms on the species richness of their flea and gamasid mite faunas. We also tested whether the probability of between-host ectoparasite sharing is related to host phylogenetic relatedness, trait similarity or geographic distance/environmental dissimilarity between their ranges. We asked whether the effects of host-associated determinants of ectoparasite richness and the probability of ectoparasite sharing differ between (1) biogeographic realms and (2) fleas and mites. Whenever significant effects of host body mass on ectoparasite richness were found, they were negative, whereas the significant effects of geographic range size, diversity field and habitat breadth were positive. The occurrence of each determinant’s effects on ectoparasite species richness differed (1) within fleas or mites between realms and (2) between fleas and mites within a realm. In all realms, the probability of a flea or a mite species being shared between hosts decreased with a decrease in the hosts’ phylogenetic relatedness, trait similarity, geographic distance between ranges or environmental similarity. The probabilities of an ectoparasite species being shared between hosts were most strongly related to the hosts’ trait similarity and were least related to the environmental similarity. We conclude that caution is needed in making judgements about the generality of macroecological patterns related to parasites based on the investigations of these patterns in limited numbers of localities and when pooling data on various taxa.

## Graphical abstract

**Figure:**
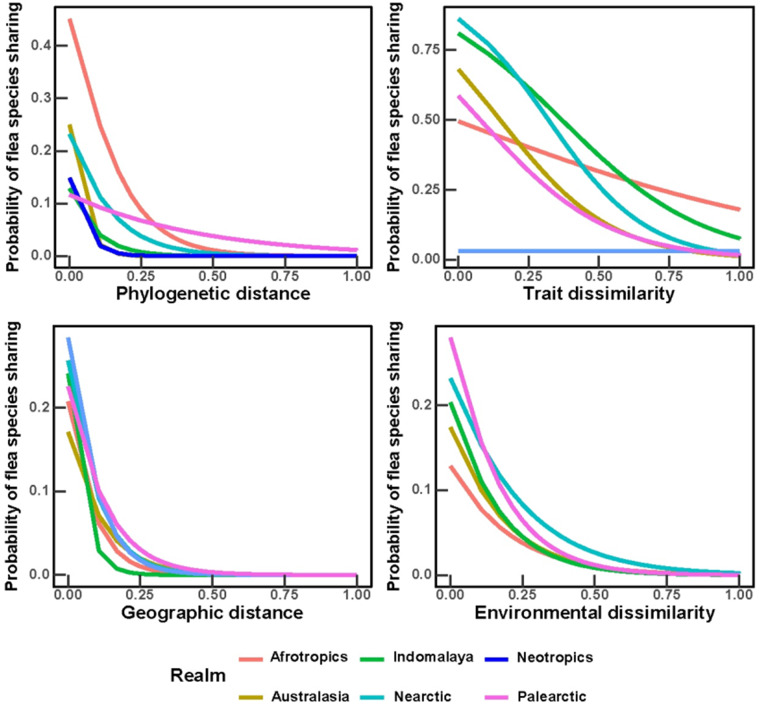

## Introduction

Parasites represent a large (if not the largest) component of global biodiversity (Poulin, 1996; Poulin and Morand, 2004; Dobson et al., 2008; Okamura et al., 2018). One of the most fundamental challenges in ecological parasitology is elucidating the determinants of parasite species richness (Poulin, 1997; Morand, 2015; Carlson et al., 2020; Dallas et al., 2020). Given that hosts represent the ultimate resource for parasites, the search for factors explaining variation in parasite species richness has mostly focused on variation in host-associated attributes (Kamiya et al., 2014; Morand, 2015). Among these attributes, parasite richness has most often been studied in relation to host body size (e.g., Morand and Poulin, 1998), density (e.g., Morand, 2000), longevity (e.g., Cooper et al., 2012a), level of sociality (e.g., Bordes et al., 2007), the number of host species cohabitating with a target host (e.g., Krasnov et al., 2004a) and geographic range size (e.g., Dáttilo et al., 2020).

The effect of some host features, such as geographic range size, on their parasites’ species richness has been found to be consistent in many host and parasite taxa, in many regions and across multiple scales (Feliu et al., 1997; Krasnov et al., 2004a; Lindenfors et al., 2007; Dáttilo et al., 2020). However, the relationships between parasite species richness and many other host traits have appeared to be variable. For example, no effect of host body size on parasite species richness was found for helminths in various terrestrial mammals (Morand and Poulin, 1998) or for fleas parasitic on rodents and shrews (Krasnov et al., 2004a), whereas this effect was found to be positive for various parasite taxa in ungulates (Ezenwa et al., 2006). Higher host assemblage species richness promoted the parasite species richness of individual host species in some, but not other, regions (Krasnov et al., 2004a vs Dáttilo et al., 2020).

The contradictory findings regarding the links between parasite species richness and host characteristics suggest that these relationships may vary between host–parasite associations. For example, Sasal et al. (1997) reported a positive association between fish body size and the species richness of monogeneans, but not of gastrointestinal helminths, explaining this difference via the differential ways fish hosts acquire ecto- and endoparasites. Other likely reasons for the above-mentioned contradictions include the study’s considered scale and geographic region. Given that parasite communities are fragmented among host individuals, populations and species, the host-associated determinants of parasite species richness have been considered at various scales, from individual hosts (infracommunities; e.g., Spickett et al., 2017) via host populations (component communities; e.g., Morand et al., 2000a) to host species (parasite faunas; e.g., Kennedy and Bush, 1992). Parasite communities at different scales substantially differ in their longevity and assembly mechanisms, with an infracommunity being ephemeral and assembled during an individual host’s lifespan via epidemiological and ecological mechanisms, whereas the persistence of a parasite fauna (i.e., a set of parasites exploiting a host species across its geographic range) is much longer, being formed during the host species’ long phylogenetic history via evolutionary processes (Poulin, 1996). From this perspective, a parasite fauna seems to be the most suitable scale for investigating the host determinants of parasite species richness, especially given that parasite infra- and component communities are obviously not appropriate for studying parasite richness in relation to some host traits, such as geographic range size. Many studies on the association between host traits and parasite species richness have been carried out at the global scale (Morand and Poulin, 1998; Nunn et al., 2003; Ezenwa *et al*., 2007; Dallas et al., 2019). To the best of our knowledge, the revealed patterns have never been compared between the same parasite–host associations from different biogeographic realms. However, parasite–host relationships in different realms have different evolutionary histories (e.g., Medvedev, 2005; Lei et al., 2024), which can cause between-realm variation in the responses of parasite species richness to the same or different host traits.

The effects of host traits on parasite communities could be realized not only via the number of parasite species but also via their identities. This is because parasites coevolved with their hosts (e.g., Brooks, 1979), adapting to species-specific host traits for the sake of successfully extracting resources from the hosts (e.g., Morand et al., 2000b). This results in similarities in parasite species composition between hosts possessing similar traits (Huang et al., 2014; Lehun *et al*., 2024). Given that many traits are usually more similar between phylogenetically close species than between phylogenetically distant species, due to a shared evolutionary history (Blomberg and Garland, 2002; Losos, 2008 and references therein), the trait-based between-host similarity in parasite species composition leads to a tight link between the similarity of parasite species composition and hosts’ phylogenetic relatedness (Poulin, 2010; Krasnov et al., 2010; Huang et al., 2014). If a parasite species can exploit a set of functionally similar and phylogenetically related hosts, it can thus alternate between these hosts, provided they spatially co-occur. In other words, a sharing of parasites between hosts is expected based on their trait and/or phylogenetic similarity (Cooper et al., 2012b; Clark et al., 2018; Dallas et al., 2019). Analogously to host determinants of parasite species richness, the probability of sharing parasites in dependence on similarity in traits or phylogenetic positions can vary geographically because of the environmental variation, biogeographic barriers and differences in the evolutionary histories of parasite–host associations (Clark et al., 2018; Gupta et al., 2019).

An additional factor that may affect a host’s parasite species richness and a parasite’s probability of being shared between hosts is the hosts’ spatial co-occurrence. First, the richer species composition of a host assemblage increases the probability of the lateral transfer of parasites and, consequently, a host’s parasite richness (Combes, 2001; but see Dáttilo et al., 2020). Second, similar parasite species compositions and the probability of parasite sharing are obviously either more probable or can only occur, respectively, between co-occurring hosts, all else being equal (Krasnov et al., 2004a; Davies and Pedersen, 2008). These patterns, again, may vary geographically.

Here, we used data on 2 taxa of arthropod ectoparasites (fleas and gamasid mites), harboured by small mammalian hosts across their geographic ranges (i.e., flea and mite faunas), from 6 biogeographic realms. First, we tested whether ectoparasite species richness correlates positively with host body mass, geographic range size, the number of co-occurring hosts within a focal host’s geographic range and the number of habitats occupied by a host. Larger hosts are expected to harbour richer parasite assemblages than smaller hosts because of their greater longevity (facilitating parasite accumulation) and larger space and higher number of niches provided for parasites (Poulin, 1995, 2004). Hosts possessing larger geographic ranges and/or occupying multiple habitats have greater chances of encountering more parasite species (Combes, 2001), whereas the reason for hosts in richer assemblages to have richer parasite fauna has been mentioned earlier. Second, we tested whether the probability of ectoparasite sharing is higher for hosts that (1) are phylogenetically close, (2) are similar in their traits, (3) are geographically close and (4) inhabit similar environments. Finally, we asked whether the results of the above-mentioned tests differ between (1) fleas and mites and (2) within fleas and mites between biogeographic realms.

## Materials and methods

### Data on fleas and gamasid mites recorded on small mammalian hosts

We used various literature sources (including many ‘grey’ publications) to obtain data on the species composition of fleas and parasitic gamasid mite harboured by small mammalian hosts (Dasyuromorphia, Paramelemorphia, Diprotodontia, Macropodiformes, Didelphimorphia, Paucituberculata, Microbiotheria, Macroscelidea, Afrosoricida, Scandentia, Notoryctemorphia, Eulipotyphla, Rodentia and the ochotonid Lagomorpha) from 6 biogeographic realms (the Afrotropics, the Australasia, the Indomalaya, the Nearctic, the Neotropics and the Palearctic) (see references for data on fleas in Krasnov et al., 2022a and on mites in Supplementary material, Appendix 1). We focused on studies that aimed to compile the most complete lists of fleas or mites on a given host species in a region or an entire continent. In total, we used data on 1090 host species infested by 1174 flea species and 884 host species infested by 643 mite species (Supplementary material, Appendix 2). Ubiquitous host species (*Mus musculus, Rattus rattus* and *Rattus norvegicus*) were not considered in the analyses.

### Host-associated determinants of ectoparasite species richness

Data on host body mass and habitat breadth (number of distinct suitable level 1 IUCN habitats) were obtained from the COMBINE database (Soria et al., 2021). Geographic host ranges were taken from Digital Distribution Maps downloaded from the IUCN database (IUCN, 2024), and a 1° × 1° cell grid was overlaid onto these maps. Then, geographic range sizes were calculated using the ‘lets.range’ function (with the ‘meters’ option) of the R package ‘letsR’ (Vilela and Villalobos, 2015). Values of geographic range size were ln-transformed prior to further analyses. To estimate the tendency of a host species to co-occur with many or a few other species (Villalobos et al., 2013), we followed Dáttilo et al. (2020) and calculated the diversity field of each host (Arita et al., 2008; Villalobos and Arita, 2010; Villalobos et al., 2013) within a respective realm. The diversity field of a species is defined as the mean number of other species that co-occur within its range. To calculate the diversity field of a focal host, we took into consideration all small mammal species that co-occurred with this host within its range, independent of whether any flea or gamasid mite was recorded on these species. Diversity fields were calculated using the function ‘lets.field’ of the ‘letsR’ package.

### Distance matrices

We constructed pairwise between-host phylogenetic, trait-based, geographic and environmental distance matrices, separately for flea and mite faunas and for each realm. Host phylogenetic trees (topology and branch lengths) were taken as 1000 random subsets from the 10°000 species-level birth-death tip-dated completed trees for 5911 mammal species of Upham et al. (2019). Consensus trees for each realm were built with the ‘consensus.edge’ function of the ‘phytools’ package (Revell, 2012), implemented in the R Statistical Environment (R Core Team, 2024). Each resulting tree was then ultrametrized using the ‘force.ultrametric’ function (with the method = ‘extend’ option) of the ‘phytools’ package, and polytomies were resolved using the ‘fix.poly’ function of the R package ‘RRphylo’ (Castiglione et al., 2018). Phylogenetic distance matrices were constructed using the ‘cophenetic.phylo’ function of the R package ‘ape’ (Paradis and Schliep, 2019).

Trait-based between-host distance matrices were based on 18 species-specific trait values, including adult body mass, relative brain mass, maximal longevity, age at first reproduction, gestation time, litter size, number of litters per year, interbirth interval (time between reproduction events), weaning age, generation length (average age of parents of the current cohort), dispersal distance (the distance an animal travels between its place of birth to the place of reproduction), hibernation or torpor (yes or no), fossoriality (ground/fossorial or above-ground dwelling), trophic level (omnivore, herbivore or insectivore), foraging stratum (ground level, scansorial or arboreal), activity cycle (nocturnal, diurnal or cathemeral), habitat breadth (number of distinct suitable level 1 IUCN habitats) and geographic range size. Values for the former 17 traits were taken from the COMBINE database (Soria et al., 2021), whereas geographic range sizes were calculated as described earlier. The values of 12 continuous traits were normalized to range from zero to unity. We constructed the trait-based distance matrices from these data using the Gower distance coefficient with the ‘gowdis’ function of the R package ‘FD’ (Laliberté and Legendre, 2010).

To build the geographic between-host distance matrices, we first overlaid a grid of 1° × 1° cells onto the distributional maps of hosts (see above), separately for each realm, and then assembled host × cell presence–absence matrices using the ‘lets.presab’ function of the ‘letsR’ package. We then determined the centroids of each species’ geographic range, using the ‘lets.midpoint’ function implemented in the ‘letsR’ package, and calculated pairwise haversine distances using the ‘geodist’ function of the R package ‘geodist’ (Padgham and Sumner, 2024).

To calculate environmental dissimilarity between the geographic ranges of host species separately for each realm, 12 environmental variables (isothermality, temperature seasonality, mean daily air temperatures of the warmest and coldest quarters, annual precipitation amount, precipitation seasonality, mean monthly precipitation amount of the warmest and coldest quarters, mean monthly climate moisture index, mean near-surface relative humidity, mean potential evapotranspiration and net primary productivity) were averaged across 1 km × 1 km grids around the centroid of a given host’s geographic range, with a 100-km buffer. These variables presumably affect ectoparasite distribution because fleas and mites are sensitive to ambient temperature and relative humidity. Environmental data were obtained from the CHELSA 2.1 datasets (Karger et al., 2017, 2021). Because of the high correlation between many of the environmental variables, we first extracted from them 2 (for the Australasia and the Palearctic) or 3 (for the remaining realms) principal components that explained from 78.84% (in the Palearctic) to 89.70% (in the Indomalaya) of the environmental variation. Then, we used the scores of these principal components to compute the classical Euclidean distance between each pair of hosts as a measure of environmental distance. For all distance matrices, the distances were normalized to range from zero to unity.

### Data analysis: determinants of ectoparasite species richness

To understand the relationships between flea or mite species richness and host body mass, diversity field (reflecting the species richness of assemblages containing a focal host), geographic range size and habitat breadth (reflecting the degree of a host’s ecological specialization), we ran generalized linear models with a negative binomial distribution and a log-link function because ectoparasite species richness is a count variable. Values of host body mass, diversity field and geographic range size were ln-transformed. Prior to running the models, we tested for phylogenetic signals in flea or mite species richness within each realm, using the K*-statistic of Blomberg et al. (2003), calculated with the ‘phyloSignal’ function of the R package ‘phylosignal’ (Keck et al., 2016). No significant phylogenetic signal was detected in any realm (Blomberg *et al*.’s K* = 0.08–0.14, *P* > 0.10 for all), and we ran models without correction for the potential confounding effect of phylogeny. The models were fitted using the ‘glm.nb’ function of the R package ‘MASS’ (Venables and Ripley, 2002). Initially, we fitted models with all possible combinations of the explanatory variables and then selected the best model based on the Akaike information criterion using the ‘model.sel’ function of the R package ‘MuMIn’ (Bartoń, 2024). The pseudo-*R*^2^ for each best model was calculated as Nagelkerke’s (1991) *R*^2^ using the ‘pR2’ function of the R package ‘modEvA’ (Barbosa et al., 2013).

### Data analysis: probability of ectoparasite species sharing

We calculated the probability of sharing ectoparasite species between hosts in dependence on their phylogenetic, trait-based, geographic and environmental distances, following Gilbert et al. (2012), Ál and Lira-Noriega (2017) and Dáttilo et al. (2020). This was done for ectoparasite species recorded on at least 6 host species. In brief, an incidence matrix with ectoparasites in rows and hosts in columns was constructed for each realm. Each host species was considered as the source of the parasite species that interacted with it and the source for a random sample of the other hosts [see details in Ál and Lira-Noriega (2017) and Dáttilo et al. (2020)]. Then, we calculated the coefficients of logistic regressions (intercept and slope) relating ectoparasite–host incidences to phylogenetic, trait-based, geographic or environmental distances between hosts as explanatory variables. This was done both for each ectoparasite species and for the entire set of ectoparasites. In the latter case, coefficients were calculated 1000 times with a random set of ectoparasite species, producing the general tendency of each coefficient distribution. Finally, the probability (*P*) of host species sharing flea or mite species in dependence on between-host phylogenetic, trait-based or geographic distances was calculated as *P* = 1/(1 + *e*^−*a*+b*D^), where *a* is the mean intercept, *b* is the mean slope and *D* is the respective between-host distance (Ál and Lira-Noriega, 2017). These analyses were carried out using the R package ‘geotax’ (Ál and Lira-Noriega, 2017) and the R functions and code compiled by ALRF.

## Results

### Determinants of ectoparasite species richness

**Figure 1.**
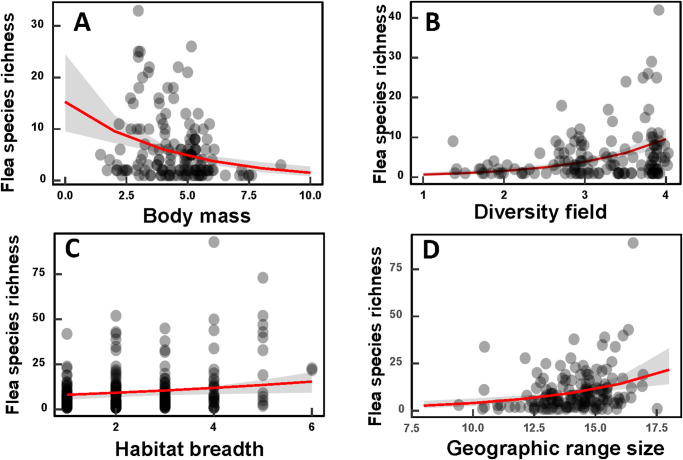
Relationships between flea species richness and (A) host body mass in the Indomalaya, (B) mean number of small mammals cohabitating with a focal host (diversity field) in the Australasia, (C) host habitat breadth in the Palearctic and (D) host geographic range in the Nearctic.

**Figure 2.**
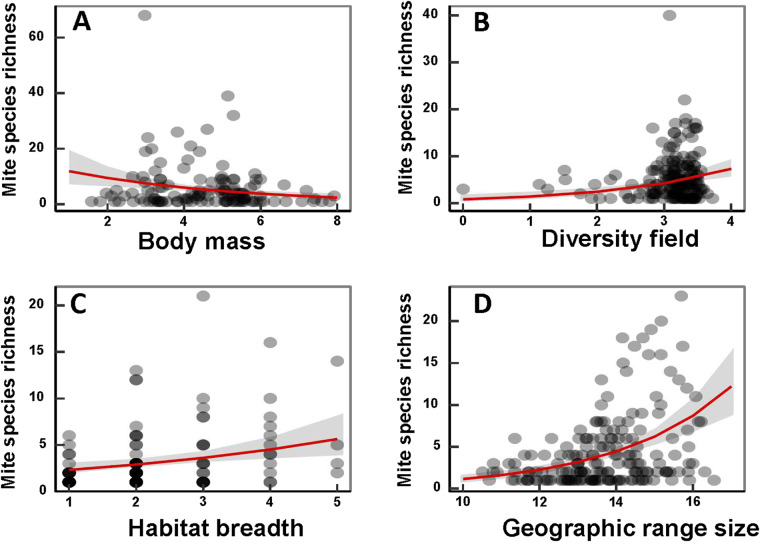
Relationships between mite species richness and (A) host body mass in the Indomalaya, (B) mean number of small mammals cohabitating with a focal host (diversity field) in the Nearctic, (C) host habitat breadth in the Australasia and (D) host geographic range in the Neotropics.

Generalized linear models demonstrated that the host-associated determinants of flea and mite species richness differed between realms (Tables 1–2). In 3 of the 6 realms, the species richness of flea and mite faunas varied between hosts depending on their body mass. Whenever the effect of host body mass on ectoparasite species richness was significant, it was negative, reflecting a lower number of flea or mite species on larger hosts (see illustrative examples for flea and mite faunas in the Indomalaya in Figure 1A and 2A, respectively). Flea and mite species richness correlated positively with host diversity field in 5 (for fleas) and 4 (for mites) realms, increasing with the number of small mammal species cohabitating with a focal host (see illustrative examples for flea faunas in the Australasia in Figure 1B and for mite faunas in the Nearctic in Figure 2B). Positive relationships between flea and mite faunas and host habitat breadth were detected in 3 and 4 realms, respectively (Tables 1–2; see illustrative examples for flea faunas in the Palearctic in Figure 1C and mite faunas in the Nearctic in Figure 2C). Host geographic range affected its flea and mite species richness in all realms, with greater numbers of flea or mite species harboured by broadly, as compared with narrowly, distributed hosts (see illustrative examples for flea faunas in the Nearctic in Figure 1D and mite faunas in the Neotropics in Figure 2D).

**Table 1.** Summary of the best generalized linear models with negative binomial distributions of the effects of host body mass (BM), geographic range size (GR), diversity field (DF; see the text for explanation) and habitat breadth (HB) on the species richness of a host’s flea fauna (FSR)

Realm	Equation FSR =	ED	*R*^2^
Afrotropics	−1.15 − 0.22 * BM + 0.23 * GR + 0.39 * DF	23.82	0.27
Australasia	−4.78 + 0.22 * GR + 0.91 * DF + 0.29 * HB	29.75	0.13
Indomalaya	−2.70 − 0.23 * BM + 0.26 * GR + 0.65 * DF	26.85	0.30
Nearctic	−1.99 + 0.21 * GR + 0.42 * DF	11.79	0.13
Neotropics	0.18 * GR + 0.15 * HB	15.47	0.17
Palearctic	−2.58 − 0.1 * BM + 0.24 * GR + 0.66 * DF + 0.13 * HB	31.68	0.38

*Note*: All coefficients are significant (*P* < 0.05).

ED, percentage of explained deviance; *R*^2^, pseudo Nagelkerke’s *R*^2^.

**Table 2.** Summary of the best generalized linear models with negative binomial distributions of the effects of host body mass (BM), geographic range size (GR), diversity field (DF; see the text for explanation) and habitat breadth (HB) on the species richness of a host’s mite fauna (MSR)

Realm	Equation MSR =	ED	*R*^2^
Afrotropics	−2.17 + 0.12*GR + 0.45*DF + 0.23*HB	21.50	0.23
Australasia	−2.06 + 1.33*GR + 0.71*DF + 0.22*HB	32.74	0.35
Indomalaya	−1.88−0.23*BM + 0.34*GR	21.92	0.24
Nearctic	−4.37 + 0.28*GR + 0.55*DF + 0.12*HB	36.30	0.44
Neotropics	−2.58−0.15*BM + 0.34*GR	23.54	0.25
Palearctic	−4.98−013*BM + 0.35*GR + 0.89*DF + 0.11*HB	54.68	0.72

*Note*: All coefficients are significant (*P* < 0.05).

ED, percentage of explained deviance; *R*^2^, pseudo Nagelkerke’s *R*^2^.

### Probability of ectoparasite species sharing

**Figure 3.**
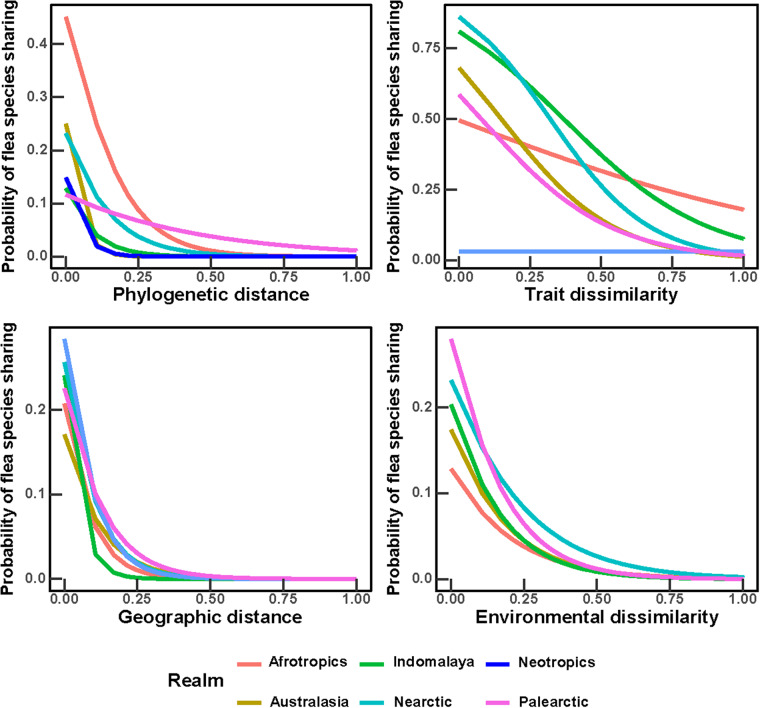
Relationships between the phylogenetic distance, trait dissimilarity, geographic distance and environmental dissimilarity between hosts and their probability to share a flea species. Lines represent mean coefficients from the logistic regressions carried out for all flea species recorded on at least 6 hosts.

**Figure 4.**
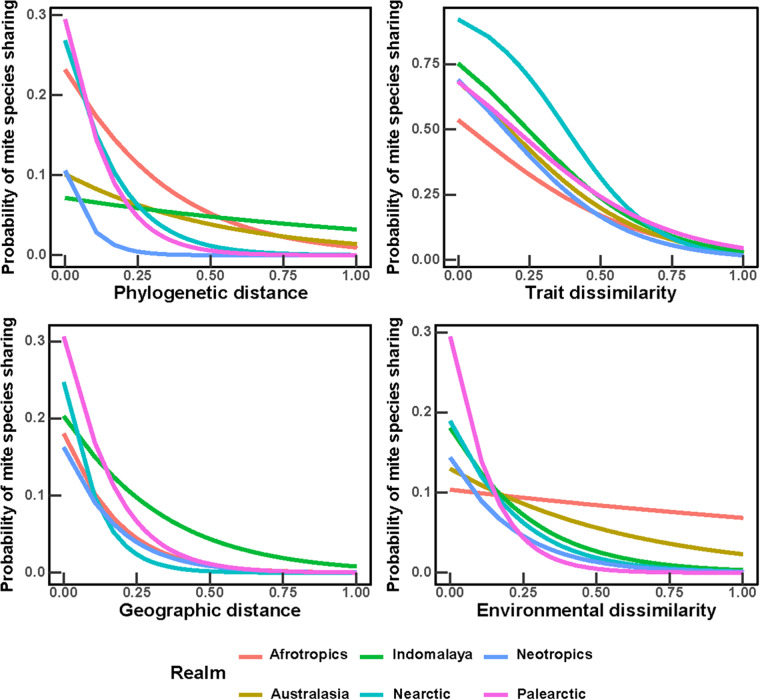
Relationships between the phylogenetic distance, trait dissimilarity, geographic distance and environmental dissimilarity between hosts and their probability to share a gamasid mite species. Lines represent mean coefficients from the logistic regressions carried out for all mite species recorded on at least 6 hosts.

The coefficients of the logistic regressions of ectoparasite–host incidences, in relation to the phylogenetic, trait-based, geographic and environmental distances between hosts, were negative in the majority of ectoparasite species, although not in all (Tables 3–4). In other words, the probability of sharing the majority of ectoparasites between hosts increased with a decrease in hosts’ phylogenetic relatedness, trait similarity, geographic distance between ranges and environmental similarity (Figures 3–4). The mean values of the intercepts and slopes of the effects of phylogenetic, trait, geographic and environmental distances differed between realms, with mean slopes being consistently negative (Tables 3–4). This resulted in somewhat different shapes of the relationships between the probability of ectoparasite sharing and its determinants in some realms, except for the effects of geographic distance, which were similar (Figures 3–4). In general, the highest probability of a flea and a mite species to be shared between hosts was related to hosts’ trait similarity (Figures 3–4), except in the Neotropics where geographic proximity played a stronger role (Figure 3). The effect of environmental similarity on the probability of ectoparasite sharing was lower than the effects of phylogenetic relatedness, trait similarity and geographic distance, except in the Palearctic where this effect was relatively strong (Figure 4).

**Table 3.** Mean values of the intercept and slope coefficient of the logistic regressions relating flea species incidences on hosts to phylogenetic (PD), trait-based (TD), geographic (GD) and environmental (ED) distances between host species and the proportion of flea species recorded on at least 6 host species with negative slope coefficients (PNS)

Realm	Number of flea species	Distance	Intercept	Slope	PNS
Afrotropics	94	PD	−0.93	−4.65	0.99
		TD	1.50	−5.64	0.94
		GD	−1.11	−7.28	1.00
		ED	−1.57	−5.14	0.97
Australasia	37	PD	−0.60	−27.50	0.47
		TD	1.25	−5.11	0.89
		GD	−1.19	−7.95	0.94
		ED	−1.12	−5.37	0.94
Indomalaya	47	PD	−1.99	−1.84	0.87
		TD	7.21	−13.11	1.00
		GD	−1.24	−15.60	1.00
		ED	−1.74	−3.63	0.87
Nearctic	93	PD	−0.73	−8.25	0.79
		TD	1.62	−5.44	0.74
		GD	−1.07	−11.43	0.97
		ED	−2.38	−0.33	0.58
Neotropics	81	PD	−1.76	−13.61	0.77
		TD	−1.04	−2.51	0.78
		GD	−0.89	−12.52	1.00
		ED	−2.00	−3.24	0.92
Palearctic	137	PD	−2.79	−1.09	0.63
		TD	0.15	−4.17	0.77
		GD	−1.24	−8.85	1.00
		ED	−2.78	−1.10	0.62

**Table 4.** Mean values of the intercept and slope coefficient of the logistic regressions relating mite species incidences on hosts to phylogenetic (PD), trait-based (TD), geographic (GD) and environmental (ED) distances between host species and the proportion of mite species recorded on at least 6 host species with negative slope coefficients (PNS)

Realm	Number of mite species	Distance	Intercept	Slope	PNS
Afrotropics	30	PD	−1.20	−3.39	0.92
		TD	0.34	−3.68	0.71
		GD	−1.51	−6.22	0.97
		ED	−2.16	−0.45	0.67
Australasia	24	PD	−2.18	−2.05	0.62
		TD	0.77	−4.32	0.71
		GD	−1.35	−3.43	1.00
		ED	−1.90	−1.84	0.82
Indomalaya	39	PD	−2.56	−0.84	0.71
		TD	1.11	−4.54	0.85
		GD	−1.17	−10.20	0.89
		ED	−1.51	−4.17	0.92
Nearctic	41	PD	−1.00	−6.84	1.00
		TD	2.45	−6.46	0.80
		GD	−1.11	−10.43	0.98
		ED	−1.46	−5.02	0.93
Neotropics	41	PD	−2.14	−12.95	0.76
		TD	0.79	−4.81	0.73
		GD	−1.64	−6.20	0.90
		ED	−1.79	−5.01	0.90
Palearctic	73	PD	−0.87	−8.50	0.97
		TD	0.76	−3.81	0.76
		GD	−0.82	−7.31	0.99
		ED	−0.87	−8.86	0.97

## Discussion

We found consistent patterns of ectoparasite species richness variation along the gradients of host body size, geographic range, diversity field and habitat specialization. The species richness of both fleas and mites was higher in smaller hosts that were broadly distributed, occurred in species-rich assemblages and occupied several habitat types. As mentioned earlier, richer parasite faunas in larger hosts are expected because of parasite accumulation due to these hosts’ longer lifespans and the greater space they provide for parasites, which may result in higher numbers of niches available for parasites (Poulin, 1995; Poulin and Morand, 2004). Positive relationships between parasite species richness and host body mass have been found in some parasite–mammal associations (Vitone et al., 2004; Ezenwa et al., 2006; Lindenfors et al., 2007), whereas in other associations, the relationship between host body mass and parasite richness was either absent (Morand and Poulin, 1998; Nunn et al., 2003; Krasnov et al., 2004a) or negative (Dáttilo et al., 2020; Villalobos-Segura et al., 2020). Studies that reported a negative association between parasite richness and host body mass noted that this pattern was mainly characteristic of small-bodied hosts, such as rodents and chiropterans; this was explained by differences in sampling efforts, with smaller hosts being more exhaustedly sampled so that the chances to record rare parasite species are higher for smaller than for larger hosts (Villalobos-Segura et al., 2020). Another explanation was that smaller mammals are usually characterized by higher population densities, causing a kind of parasite species ‘dilution’ among host species (Dáttilo et al., 2020). Here, we propose an additional explanation for the negative association between host body mass and parasite richness found in our study. As mentioned earlier, the parasite taxa under consideration (fleas and gamasid mites) are predominantly nidicolous, and most of their lives are spent in their hosts’ burrows and nests (Radovsky, 1985; Krasnov, 2008). Furthermore, the pre-imaginal development of the absolute majority of flea species takes place in hosts’ burrows/nests (Krasnov, 2008). Smaller mammals usually construct deep burrows, with more complex architecture than those of larger mammals (even within a 2.5–5000-g mass range, which is the definition for a ‘small mammal’; Degen, 1997) (Kucheruk, 1983). These burrows represent hotspots of flea and mite diversity (Holland, 1964), leading to an increase in flea and/or mite species richness in the burrows’ owners (Krasnov et al., 2004b).

Positive relationships between flea and mite species richness and host geographic range size and/or the number of occupied habitats are not especially surprising. The effect of host geographic range size and habitat generalism has been repeatedly shown for various parasites and host taxa (e.g., Morand, 2000, 2015; Torres et al., 2006; Costello, 2016). The most likely mechanism behind this pattern is that hosts that have large geographical ranges or persist in many habitats accumulate large numbers of parasite species because of their higher probabilities to encounter many parasite species (Combes, 2001; Morand, 2015). In addition, a larger geographic range and/or habitat generalism likely results in higher probabilities to encounter many other host species, which might facilitate the between-host exchange of parasites. In the case of nidicolous ectoparasites, this exchange may be realized via visiting each other’s burrows (Krasnov, 2008) or via direct contact between individual hosts belonging to different species (Krasnov and Khokhlova, 2002). As a result, parasite species richness increases in broadly distributed, habitat-generalist hosts occurring in species-rich host assemblages (measured via the mean number of cohabitating hosts, i.e., diversity field), as was found in our study [see also Krasnov et al. (2004a) for fleas in a subset of 92 Holarctic hosts]. However, Dáttilo et al. (2020) found a positive effect of host geographic range on ectoparasite species richness in mammals in Mexico, but no relationship between ectoparasite richness and host diversity field. This contradiction between our results and those of Dáttilo et al. (2020) may be associated with differences in the scale of the analyses (a biogeographic realm versus a single country), as well as with the fact that we considered 2 distinct taxa of ectoparasites feeding either obligatorily or facultatively on host blood, while Dáttilo et al. (2020) considered data pooled on all ectoparasite taxa, using data from Whitaker and Morales-Malacara (2005). The latter included lice (Phthiraptera), which are strictly host-specific (e.g., Light et al., 2010) and usually do not switch host species, as well as a variety of phoretic, predatory and saprophagous arthropods that are not parasitic.

Furthermore, we found that ectoparasite species richness was driven by host-associated variables differently (1) in different biogeographic realms and (2) between fleas and mites within the same realm. For example, the host diversity field did not explain either flea or mite species richness in the Neotropics, but it did so in other realms (for at least 1 of the 2 ectoparasite taxa) (Tables 1–2). Host body mass predicted flea species richness in the Afrotropical, Indomalayan and Palearctic, but not in the Australasian, Nearctic and Neotropical hosts. The effect of host body mass on mite species richness was detected in the Australasia, Neotropics and Palearctic, but not in the Afrotropics, Indomalaya or the Nearctic. Flea vs mite differences can be exemplified by the effect of host body mass on flea, but not mite, species richness in the Afrotropics or the effect of host habitat breadth on mite, but not flea, species richness in the Nearctic. One of the most likely reasons for these differences is between-realm differences in the species compositions of hosts, fleas and mites that resulted from the differential histories of hosts, parasites and their interactions (e.g., Medvedev, 2005; Zhu et al., 2015 for fleas). The responses of host-associated variables may vary between different flea or mite species, leading to the between-realm variation in the host drivers of parasite species richness. Moreover, the between-realm variation in the average degree of flea or mite host specificity might also cause differences in the relationships between their species richness and host-associated predictors. For example, flea–host interactions in the Palaearctic appeared to be relatively more specialized than those in the Nearctic, resulting in each flea species interacting with fewer host species in the former than in the latter (Krasnov et al., 2007). This might be one of the reasons behind the effect of host body mass on flea species richness in the Palearctic but not in the Nearctic. The difference between the predictors of flea and mite richness in the same realm could be somehow associated with the differential life histories of these taxa. In particular, fleas are obligate haematophages, but their pre-imagoes (except for a few species) are not parasitic, whereas in many mites, the pre-imagoes are also blood-feeding, but some species only feed on a host’s blood facultatively. The level of mite host specificity is much lower than that of fleas (Vinarski et al., 2007). These life history differences have been proposed as factors that may cause differences between the 2 taxa in a number of ecological and biogeographic patterns within the same biogeographic realm (Krasnov et al., 2004a vs Korallo et al., 2007; Krasnov et al., 2005 vs Vinarski et al., 2007).

We found that the probability of a flea or a mite species being shared between hosts decreased with an increase in the between-host phylogenetic distance, trait dissimilarity, geographic distance and environmental dissimilarity. The most likely reasons behind the effect of phylogenetic relatedness and trait similarity are that (1) ectoparasites select hosts with traits that allow parasites to successfully extract resources from these hosts (Krasnov et al., 2016) and (2) phylogenetically close relatives are often more similar to each other than distant relatives are (phylogenetic trait conservatism; Blomberg and Garland, 2002; Losos, 2008). In addition, a parasite may originate on a given host and then does not speciate following its host’s speciation, resulting in the same parasite being present on multiple daughter lineages of the original host (a so-called ‘inertia event’) (Paterson and Banks, 2001). Nevertheless, trait resemblance appeared to be the most important factor facilitating ectoparasite sharing, with the negative effect of host trait dissimilarity on ectoparasite sharing being much stronger than that of host phylogenetic distance (except for the Neotropical fleas). This is counterintuitive given the above-mentioned phylogenetic trait conservatism. However, phylogenetic trait conservatism may not always be the case, especially for cohabitating close relatives. Limiting similarity theory (MacArthur and Levins, 1967) states that interspecific competition increases with an increase in niche similarity, ultimately leading to co-occurring species possessing dissimilar niches, which in turn, represents an outcome of trait differences. Assuming trait similarity between close relatives, one of the predictions of this theory is that competition exclusion results in the unlikelihood of the co-occurrence of closely related species (e.g., Webb et al., 2002). However, Mayfield and Levine (2010) demonstrated that, in some cases, competition can lead to the exclusion of less related species. This can be due to either trait similarity in distant relatives or no association between trait similarity and phylogenetic relatedness (e.g., Uriarte et al., 2010).

The higher probability of ectoparasite sharing being more strongly determined by trait similarity than by phylogenetic relatedness may be the result of the process known as ‘ecological fitting’ (Janzen, 1985). Ecological fitting represents a situation in which an organism (e.g., a parasite) interacts with its environment (e.g., a host) in a way that might suggest a shared evolutionary history, whereas in reality, the traits relevant to the interaction evolved elsewhere and in response to a different set of conditions. When parasites depend on the resource rather than a specific host, and when phylogenetically distant hosts share this resource, host-switching becomes likely. A new host may be unrelated to the parasite’s original host species (Brooks et al., 2006; D’Bastiani et al., 2023). In the case of nidicolous ectoparasites, a necessary resource possessed by many related and unrelated host species is their burrows/nests where (1) the majority of fleas and mites spend the main part of their lives and (2) most pre-imaginal development occurs. In the Neotropical hosts, however, the probability of sharing a flea species, but not a mite species, was almost equally determined by phylogenetic relatedness and trait similarity. This might somehow be associated with the fact that the Neotropical flea–host associations have a longer evolutionary history than those in the remaining realms. This is because fleas most likely originated in Gondwana (the former South America and Australia connected via Antarctica until the late Eocene) became first associated with aboriginal hosts and then dispersed with their hosts from the Laurasian North America (periodically connected with South America via a Caribbean land bridge during the Late Cretaceous) (Zhu et al., 2015).

The effect of geographic distance between host ranges on the probability of ectoparasite sharing might most likely be associated with either a higher probability of cohabitating hosts to harbour the same parasites (e.g., Davies and Pedersen, 2008) or the above-mentioned ecological fitting or both. Environmental similarity could result in similar microclimatic conditions in the burrows of different host species (Degen, 1997). These similar conditions would allow nidicolous arthropods sensitive to air temperature and relative humidity (Marshall, 1981) to inhabit these burrows and exploit their owners.

In conclusion, the host-associated determinants of the probability of sharing ectoparasite species were, in general, similar between (1) fleas and mites and (2) geographic realms. However, this was not the case for the determinants of the species richness of flea and mite faunas. Therefore, our findings indicate that caution is warranted when generalizing macroecological patterns in parasites – particularly when such patterns are inferred from studies with limited geographic scope or by pooling data across diverse parasite taxa. Our results demonstrate that evolutionary contingencies – shaped by regional biogeographic histories and parasite life-history strategies – can override large-scale ecological predictions. Consequently, studies extrapolating parasite macroecological patterns from restricted taxonomic or geographic sampling risk oversimplifying these complex biological systems.

Future research should employ comparative frameworks incorporating phylogenetic and biogeographic contexts to advance a more robust, nuanced understanding of parasite biodiversity patterns.

## Supporting information

Krasnov et al. supplementary material Krasnov et al. supplementary material

## Data Availability

Raw data on flea and host species at the scale of biogeographic realms are contained in the sources cited in Krasnov et al. (2022a) and the Supplementary material. Custom R functions and the R Code can be obtained from A.L.R.-F. (a474r867@ku.edu) upon request.

## Supplementary material

The supplementary material for this article can be found at https://doi.org/10.1017/S0031182025100371.
