# Supplementary material for: Geographic variation in the determinants of ectoparasite faunas’ species richness: fleas and gamasid mites parasitic on small mammals from 6 biogeographic realms
Source: Parasitology. 2025 Jun 27;152(7):745–56. doi: 10.1017/S0031182025100371 (PMC12418288; doi:10.1017/S0031182025100371)
Supplement: Krasnov et al. supplementary material [file S0031182025100371sup001.docx]

**Appendix 1. Supplementary references.**

List of sources for distribution of gamasid mites on small mammalian hosts

**Abba,AM, Udrizar Sauthier DE, Bender JB, Lareschi M** (2001) Mites (Acari: Laelapidae) associated with sigmodontinae rodents in Entre Rios Province, Argentina. *Memórias do Instituto Oswaldo Cruz* **96**, 1171-1172.

**Acari Collection & Systematics**. Royal Museum for Central Africa, Tervuren (Belgium). <https://www.africamuseum.be/research/collections_libraries/biology/collections/acari/browse_collector?filter=&orderby=Collector&cur_page=899> (Accessed on 15-02-2025).

**Adrus M, Jazman NAM, Azizi RNAR, Ahamad M, Tajuddin AM** (2021) Ectoparasites fauna of rodents and scandents at different habitats of Sarawak, Malaysia. *Serangga* **26**, 26-46.

**Ahmad NII, Rahim NAA, Roslan A, Adrus M, Ahamad M, Hassan M, Safiih LM, Ramlee M, Zahidin M, Abdullah, MT** (2020) Data on ectoparasites infestation on small mammals from different habitats in east-coast peninsular Malaysia. *Data in Brief* **30**, 105621.

**Airoldi JP, Solomon L, Duca AV** (1989) Les Gamasides (Acari) des nids de la forme fouisseuse du campagnol terrestre *Arvicola terrestris* L. *Revue suisse de zoologie* **96**, 161-189.

**Allred DM** (1956) Mites found on mice of the genus *Peromyscus* in Utah. I. General infestation. *The Great Basin Naturalist* **16**, 23-31.

**Allred DM** (1957a) Mites found on mice of the genus *Peromyscus* in Utah. III. Family Dermanyssidae. *The American Midland Naturalist* **57**, 450-460.

**Allred DM** (1957b) Mites found on mice of the genus *Peromyscus* in Utah. V. Trombiculidae and miscellaneous families. *The Great Basin Naturalist* **17**, 95-102.

**Allred DM** (1957c) Notes on the life history and bionomics of the wood rat mite, *Brevisterna utahensis* (Acarina). *Transactions of the American Microscopical Society* **76**, 72-78.

**Allred DM** (1958) Mites found on mice of the genus *Peromyscus* in Utah. IV. Families Laelaptidae and Phytoseiidae. *The Pan-Pacific Entomologist* **34**, 17-32.

**Allred DM** (1961) Parasitic mites on marmots in Utah. *Journal of Parasitology* **47**, 124.

**Allred DM** (1962a) Mites on grasshopper mice at the Nevada atomic test site. *The Great Basin Naturalist* **22**, 101-104.

**Allred DM** (1962b) Mites on squirrels at the Nevada atomic test site. *Journal of Parasitology* **48**: 817.

**Allred DM** (1963) Mites from pocket mice at the Nevada test site. *Proceedings of the Entomological Society of Washington* **65**, 231-233.

**Allred DM** (1966) Unusual records of Utah mites. *The Great Basin Naturalist* **26**, 34.

**Allred DM** (1969a) Haemogamasid mites of Eastern Asia and the western Pacific with a key to the species. *Journal of Medical Entomology* **6**, 103–119.

**Allred DM** (1969b) Mites of the genus *Laelaps* of New Guinea (Acari: Mesotrigmata: Laelapidae). *Journal of Medical Entomology* **6**, 337–385.

**Allred DM** (196c9) New mesostigmatid mites from Pakistan with keys to genera and species. *Journal of Medical Entomology* **6**, 219–244.

**Allred DM** (1970a) Dermanyssid mites of New Guinea. *Journal of Medical Entomology* **7**, 242-246.

**Allred DM** (1970b) Mites of the genus *Laelaps* from Viet Nam and Laos and a key to species of Indochina and Thailand. *Journal of Medical Entomology* **7**, 247-250.

**Allred DM** (1970c) New Ameroseiid mites from birds of New Guinea. *Journal of Medical Entomology* **7**, 99-102.

**Allred DM** (1970d) Two new mites (Laelapidae) from West Pakistan. *Journal of Medical Entomology* **7**, 107-111.

**Allred DM, Beck DE** (1966) Mites of Utah mammals. *Brigham Young University Science Bulletin, Biological Series* **8**, 1-123.

**Allred DM, Goates MA** (1964a) Mites from mammals at the Nevada test site. *The Great Basin Naturalist* **24**, 71-73.

**Allred DM, Goates MA** (1964b) Mites from woodrats at the Nevada nuclear test site. *Journal of Parasitology* **50**, 171.

**Allred DM, Roscoe EJ** (1957) Parasitic mites in desert wood rat nests with notes оn free-living forms. *Transactions of the American Microscopical Society* **76**, 389-403.

**Allymehr M, Tavassoli M, Manoochehri MH, Ardavan D** (2012) Ectoparasites and gastrointestinal helminths of house mice (*Mus musculus*) from poultry houses in northwest Iran. *Comparative Parasitology* **79**, 283-287.

**Ambros M** (1984) Mesostigmatic mites (Acarina) from small mammals in the Čergov Mountains (Western Carpathians Czechoslovakia). *Miscellanea Zoologica Hungarica* **2**, 35-38.

**Anderson PC and Kok OB** (2003) Ectoparasites of springhares in the Northern Cape Province, South Africa. *South African Journal of Wildlife Research* **33**, 23–32.

**AntonenkoVV** (1970) Seasonal changes in the population dynamics of *Hirstionyssus isabellinus* Oudemans, 1913 and *Hyperlaelaps arvalis* Zachvatkin, 1948 on the common vole (*Microtus arvalis* Pall*.*) *Medical Parazitology and Parasitic Diseases* **39**, 741-742 (in Russian).

**Archer EK, Bennett NC, Ueckermann EA, Lutermann, H** (2014) Ectoparasite burdens of the common mole-rat (*Cryptomys hottentotus hottentotus*) from the Cape Provinces of South Africa. *Journal of Parasitology* **100**, 79-84.

**Arnold W, von Lichtenstein A** (1993) Ectoparasite loads decrease the fitness of alpine marmots (*Marmota marmota*) but are not a cost of sociality. *Behavioral Ecology* **4**, 36-39.

**Babyesiza WS, Mpagi J, Ssuuna J, Akoth S, Katakweba A** (2023) Ectoparasite fauna of rodents and shrews with their spatial, temporal, and dispersal along a degradation gradient in Mabira Central Forest Reserve. *Journal of Parasitology Research* **2023**, 7074041.

**Baker EW, Traub R, Evans TM** (1962) Indo-Malayan *Haemolaelaps* with description of new species (Acarina: Laelaptidae). *Pacific Insects* **4**, 91-100.

**Balashov YS** (1982) *Host-Parasite Relations Between Arthropods and Terrestrial Vertebrates.* Leningrad: Nauka (in Russian).

**Balashov YS** (2004) Structure of parasitic arthropod communities in forest small mammals. *Parazitologiia* **38**, 481-491 (in Russian).

**Balashov YS, Bochkov AV, Vashchenok VS, Grigorieva LA, Tret'iakov KA** (2002) Structure and seasonal dynamics of the biotic community ectoparasites of the bank vole in the Ilmen-Volkhov lowland. *Parazitologiia* **36**, 433-46 (in Russain).

**Balashov YS, Bochkov AV, Vashchenok VS, Tret'iakov KA** (2003) Structure and seasonal dynamics of an ectoparasite community on the common shrew (*Sorex araneus*) in the Il'men'-Volkhov lowland. *Parazitologiia* **37**, 441-454 (in Russian).

**Balashov YS, Bochkov AV, Vashchenok VS, Grigorieva LA, Staniukovich MK, Tret'iakov KA** (2007a) Structure of populations and ecological nishes of ectoparasites in the parasite communities of small forest mammals. *Parazitologiia* **41**, 329-347 (in Russian).

**Balashov YS, Bochkov AV, Vashchenok VS, Grigorieva LA, Staniukovich MK, Tret'iakov KA** (2007b) Segregation of ectoparasitic communities of small forest mammals among ecological niches. *Doklady Biological Sciences* **415**, 280-283.

**Bandyopadhyay P, Karmakar K, Halliday B** (2023) Checklist of Indian mites in the family Laelapidae (Acari: Mesostigmata). *Zootaxa* **5249**, 401–424. <https://doi.org/10.11646/zootaxa.5249.4.1>

**Banks N** (1905) Descriptions of some new mites. *Proceedings of the Entomological Society of Washington* **7**: 133-142.

**Barros DM, Linardi PM, Botelho JR** (1993) Ectoparasites of some wild rodents from Parana State, Brazil. *Journal of Medical Entomology* **30**, 1068-1070.

**Basolo F, Funk R** (1974) Ectoparasites from *Microtus ochrogaster*, *Peromyscus leucopus*, and *Cryptotis parva* in Coles County, Illinois. *Transactions of the Illinois State Academy of Science* **67**, 211-221.

**Bassini-Silva R, Jacinavicius FC, Huang-Bastos M, Dowling APG, Barros-Battesti DM** (2021) A checklist of macronyssid species (Mesostigmata: Macronyssidae) from Brazil. *Journal of Medical Entomology* **58**: 625-633.

**Beliaev VG** (1963) To the fauna of ectoparasites of Magadan Oblast. *Proceedings of the Irkutsk Plague Control Institute* **5**, 180-185 (in Russian).

**Bengtson SA, Brinck-Lindroth G, Lundqvist L, Nilsson A, Rundgren S** (1986) Ectoparasites on small mammals in Iceland: Origin and population characteristics of a species-poor insular community. *Holarctic Ecology* **9**, 143–148.

**Bengtson SA, Brinck-Llindroth G, Lundqvist, L, Nilsson, A, Rundgren, S** (1986) Ectoparasites on small mammals in Iceland - origin and population characteristics of a species-poor insular community. *Holarctic Ecology* **9**, 143-148.

**Berdyev A, Amanguliev A, Babaev J, Berdyev AS, GlebezdinVS, Erokhin PI, Meledzhaeva MA** (1987) Species composition of the fauna of parasite coenoses of gerbils. *Parazitologiia* **25**, 620-627 (in Russian).

**Berendiaeva EL** (1958) To the fauna of gamasid ticks of Frunze Oblast. *Transactions of the Central Asian Scientific Plague Control Institute* **4**, 275-277 (in Russian).

**Bespyatova LS** (2009) Species diversity and biocenotic relatioships of gamasid mites (Parasitiformes, Gamasoidea) in the nests of the field vole *Microtus agrestis L.* of the Karelian middle taiga subzone. *Entomological Review* **89**, 993-1000.

**Bhat HR, Kulkarni SM, Mishra AC** (1983) Records of Mesostigmata, Ereynetidae and Pterygosomidae (Acarina) in Western Himalayas, Sikkim and Hill Districts of West Bengal. *Journal of the Bombay Natural History Society* **80**, 91–110.

**Bibikova VA** (1959) To the fauna of trombiculid and gamasid mites of Tarbagatai. *Transactions of the Central Asian Scientific Plague Control Institute* **6**, 285-290 (in Russian).

**Bitam I** (2012) Vectors of rickettsiae in Africa. *Ticks and Tick Borne Diseases* **3**, 382-386.

**Bittencourt EB, Rocha CF** (2003) Host-ectoparasite specificity in a small mammal community in an area of Atlantic Rain Forest (Ilha Grande, State of Rio de Janeiro), Southeastern Brazil. *Memórias do Instituto Oswaldo Cruz* **98**, 793-798.

**Bochkov AV, Abramov AV, Durden LA, Apanaskevich DA, Stekolnikov AA, Stanyukovich MK, Gnophanxay S, Tikhonov AN** (2011) Arthropod symbiotes of *Laonastes aenigmamus* (Rodentia: Diatomyidae). *Journal of Parasitology* **97**, 352-353.

**Bogdanov II** (1975) Ticks on lemmings in the eastern Taimyr. *Parazitologiia* **9**, 522-525 (in Russian).

**Bogdanov II** (1979) Gamasoid mites of the Taimyr Peninsula. *Parazitologiia* **8**, 474-482 (in Russian).

**Bogdanov, II** (1983) Faunistic complexes of ticks and mites (Parasitiformes) and fleas (Siphonaptera) associated with voles of the genus *Clethrionomys* in West Siberia. *Parazitologiia* **17**, 140-148 (in Russian).

**Borisova VI, Nazarova IV** (1981) Fauna and ecology of gamasid mites of shrews from the southern taiga outpost in the Volga-Kama Territory (Acarina, Gamasoidea). *Parazitologiia* **15**, 21-26 (in Russian).

**Borisova VI, Nazarova IV** (1986) Gamasid mites of common voles in the central Volga River region. *Parazitologiia* **20**, 208-213 (in Russan).

**Botelho JR, Linardi PM, Williams P, Nagem RL** (1981) Alguns hospedeiros reais de ectoparasitos do município de Caratinga, Minas Gerais, Brasil. *Memórias do Instituto Oswaldo Cruz* **76**, 57-59.

**Botelho JR, Williams P** (1980) Sobre alguns ectoparasitos de roedores silvestres do município de caratinga, Minas Gerais, Brasil: II. Acarofauna.

**Bregetova NG** (1956) *Gamasid Mites (Gamasoidea). A Brief Identification Guide.* Moscow-Leningrad: Academy of Sciences of the USSR (in Russian).

**Capri JJ, Mauri RA** (1971) Ectoparásitos (Suctoria y Acarina) de roedores de la Familia Caviidae en Argentina. *Revista De La Sociedad Entomologica Argentina* **3**, 1-4.

**Changbunjong T, Jirapattharasate C, Buddhirongawatr R, Chewajon K, Charoenyongyoo P, Suwanapakdee S, Waengsothorn S, Triwitayakorn K, Chaichoun K, Ratanakorn P** (2010) Ectoparasitic fauna of birds, and volant and non-volant small mammals captured at Srinakarin Dam, Kanchanaburi, Thailand. *Southeast Asian Journal of Tropical Medicine and Public Health* **41**, 526-535.

**Chuluun B, Mariana A, Ho T, Mohd Kulaimi, B** (2005) A preliminary survey of ectoparasites of small mammals in Kuala Selangor Nature Park. *Tropical Biomedicine* **22**, 243-247.

**Cicek H, Stanyukovich M, Yağci S, Aktaş M, Karaer Z** (2008) Gamasine mite (Parasitiformes: Mesostigmata) infestations of small mammals (Mammalia: Rodentia, Insectivora) in Turkey. *Türkiye Parazitoloji Dergisi* **32**, 65–70.

**Coffee GM, Retief LA** (1972) The *Laelaps muricola* Complex in the Ethiopian Region; Description of a New Species and a New Subspecies (Acarina: Mesostigmata). *Journal of Medical Entomology* 9, 417–424.

**Colombo VC, Lareschi M, Monje LD, Antoniazzi LR, Morand S, Beldomenico PM** (2023) Ecological factors shaping the ectoparasite community assembly of the azara's grass mouse, *Akodon azarae* (Rodentia: Cricetidae). *Parasitology Research* **122**, 2011-2021.

**Costa M** (1961) Mites associated with rodents in Israel. *Bulletin of the British Museum (Natural History*). *Zoology* **8**, 1–70.

**Costa M** (1968) Little known and new litter-inhabiting laelapine mites (Acari, Mesostigmata) from Israel. *Israel Journal of Zoology* **17**, 1-30.

**Costa M** (1971) Mites of the genus *Pachylaelaps* Berlese (Acari: Mesostigmata, Pachylaelaptidae) from litter in Israel. *Israel Journal of Ecology and Evolution* **20**, 253-277.

**Daniel M** (1983) Mesotigmatid mites of small mammals from the Hindu Kush (Afghanistan). *Folia Parasitologica* 24, 353–366.

**Davydova MS** (1962) To the ecology of *Laelaps muris* in West Siberia] Voprosy Ecologii **8**, 43-44 (in Russain).

**Davydova MS, Belova OS** (1972) Fauna of gamasid mites of the floodplain of the Ob' River. In: Maximov AA, ed. *Biological resources of the Ob’ River floodplain*. Novosibirsk: Nauka Publishers, pp. 306–324. (In Russian).

**Davydova MS**, ed (1980) *Parasitic insects and mites of Siberia*. Novosibirsk: Nauka Publishers. (In Russian)

**Davydova MS, Nikolsky VV** (1986) *Gamasid mites of Western Siberia*. Novosibirsk: Nauka Publishers. (In Russian)

**De Oliveira GMB, Bassini-Silva R, Huang-Bastos M, Pereira JS, Sponchiado J, Guimarães MF, Dowling APG, Labruna MB, Barros-Battesti DM, Jacinavicius FC, Horta MC (2020)** Contribution about the knowledge of *Lepronyssoides pereirai* (Fonseca, 1935) (Mesostigmata: Macronyssidae): hosts and distribution. *International Journal of Acarology* **46**, 377-379.

**Delfinado MD** (1960) On some parasitic laelaptoid mites (Acarina) of the Philippines. *Fieldiana (Zoology)* **42**, 93–114.

**Desser SS** (1990) Tissue "cysts" of Hepatozoon griseisciuri in the grey squirrel, *Sciurus carolinensis*: The significance of these cysts in species of Hepatozoon. *Journal of Parasitology* **76***,* 257-259.

**Domrow R** (1961). New and little known Laelaptidae, Trombiculidae and Listrophoridae (Acarina) from Australasian mammals. *Proceedings of the Linnean Society of New Southern Wales* **86**, 60–95.

**Domrow R** (1962) Seven new species of *Laelaps* from Malaysia (Acarina, Laelaptidae. *Acarologia* **4**, 503-519.

**Domrow R** (1963) New records and species of Austromalayan laelapid mites. *Proceedings of the Linnean Society of New South Wales.* **88***,* 199-220.

**Domrow R** (1963). New records and species of Austromalayan laelapid mites. *Proceedings of the Linnean Society of New Southern Wales* **88**, 199–220.

**Domrow R** (1965). The genus *Laelaps* in Australia (Acarina: Laelapidae). *Journal of the Entomological Society of Queensland* **4**, 18–23.

**Domrow R** (1972) Two new species of *Haemolaelaps* Berlese (Acari: Dermanyssidae) from Leadbeater's possum. *Australian Journal of Entomology.* **11**, 290-294.

**Domrow R** (1973). New records and species of *Laelaps* and allied genera from Australasia (Acari: Dermanyssidae). *Proceedings of the Linnean Society of New Southern Wales* **98,** 62–85.

**Domrow R** (1976) Oriental mesostigmata (Acari) 3. A new species of Eechinonyssus from Selangor. *Oriental Insects* **10**, 179-183.

**Domrow R** (1977). New records and species of *Laelaps* and allied genera from Australasia (Acari: Dermanyssidae). Part 2. *Proceedings of the Linnean Society of New Southern Wales* **101,** 185–217.

**Domrow R** (1987) Acari Mesostigmata parasitic on Australian vertebrates: An annotated checklist, keys and bibliography. *Invertebrate Taxonomy* **1**, 817-948.

**Domrow R** (1988) Acari Mesostigmata Parasitic on Australian Vertebrates: an Annotated Checklist, Keys and Bibliography. *Invertebrate Taxonomy* **1**, 817–948.

**Domrow R** (1992) Acari Astigmata (excluding feather mites) parasitic on Australian vertebrates: an annotated checklist, keys and bibliography. *Invertebrate Taxonomy* **6**, 1459–1606.

**Dowling AP, Bochkov AV, O'Connor BM** (2007) Revision of the genus *Andreacarus* (Acari: Laelapidae) with description of seven new species and a new genus for Australian species formerly placed in Andreacarus. *Journal of Medical Entomology* **44**, 405-421.

**Dubinin VB, Dubinina MN** (1951) The parasite fauna of mammals of the Dauria steppe. In Formozov, AN (ed). *Fauna and Ecology of Rodents.* Moscow: Moscow Society of Naturalists, pp. 98-156 (in Russian).

**Durden LA, Page BF** (1991) Ectoparasites of commensal rodents in Sulawesi Utara, Indonesia, with notes on species of medical importance. *Medical and Veterinary Entomology* 5, 1-7.

**Durden LA, Banks CW, Clark KL, Belbey BV, Oliver JH** (1997) Ectoparasite fauna of the eastern woodrat, *Neotoma floridana*: Composition, origin, and comparison with ectoparasite faunas of western woodrat species. *Journal of Parasitology* **83**, 374-381.

**Durden LA, Ellis BA, Banks CW, Crowe JD, Oliver JH** (2004) Ectoparasites of gray squirrels in two different habitats and screening of selected ectoparasites for bartonellae. *Journal of Parasitology* **90**, 485-489.

**Dusbábek F, Daniel M, Til WM** (1982) Laelapidae (Acarina) of some small mammals from Toro game reserve, Uganda. *Folia Parasitologica* **29**, 167–176.

**Edler A and Mehl R** (1972) Mites (Acari, Gamasina) from Small Mammals in Norway. *Norsk Entomologisk Tidsskrift* **19**, 133–147.

**Elistratova NP** (1963) To the ectoparasite fauna of the west part of the Saylyugem Mountains. *Proceedings of the Irkutsk Plague Control Institute* **5**, 170-176 (in Russian).

**Emelianov PF, Briukhanova LV, Reitblat AG, Glushko NV, Tiflova LA** (1974) The parasite fauna of burrows of the little ground squirrel in isolated colonies in Stavropol Krai. In Pilipenko, VG (ed). *Highly Dangerous Infections in the Caucasus, Issue 1.* Stavropol: USSR Ministry of Health, pp. 140-142 )in Russian).

**Emelianova ND, Zhovtyi IF** (1957) A brief review of mammal ectoparasites of the Mongol-Transbaikal plague hotbed in the context of their epizootological significance. *Transactions of the Irkutsk State Plague Control Scientific Institute of Siberia and Far East* **15**, 259-283 (in Russian).

**Engelbrecht A, Matthee CA, Ueckermann EA, Matthee S** (2014) Evidence of cryptic speciation in mesostigmatid mites from South Africa. *Parasitology* **141**, 1322–1332.

**Espinoza-Carniglia M, Galliari C, Fantozzi MC, Beldomenico PM, Lareschi M** (2023) An integrative approach to explore species limits in *Laelaps mazzai* Fonseca, 1939 (Mesostigmata, Laelapidae), a South American widespread mite parasitizing the cricetid *Calomys* Waterhouse, 1837. *Acta Tropica* **240**, 106836.

**Evans GO, Till WM** (1966) Studies on the British Dermanyssidae (Acari: Mesostigmata). Part II. Classification. *Bulletin of the British Museum (Natural History*). *Zoology* **14**, 109–370.

**Fagir DM, Ueckermann EA, Horak IG, Bennett NC, Lutermann H** (2014) The Namaqua rock mouse (*Micaelamys namaquensis*) as a potential reservoir and host of arthropod vectors of diseases of medical and veterinary importance in South Africa. *Parasites and Vectors* **7**, 366. <https://doi.org/10.1186/1756-3305-7-366>

**Fagir DM, Horak IG, Ueckermann EA, Bennett NC, Lutermann H** (2015) Ectoparasite diversity in the Eastern Rock Sengis (*Elephantulus myurus*): the effect of seasonality and host sex. *African Zoology* **50**, 109–117.

**Fagir DM, Bennett NC, Ueckermann EA, Howard A, Hart DW** (2021) Ectoparasitic community of the Mahali mole-rat, *Cryptomys hottentotus mahali*: Potential host for vectors of medical importance in South Africa. *Parasits and Vectors* **14**, 24.

**Fedorova LV** (1957) To the ectoparasite fauna of Kabansk District of Buryat-Mongol Autonomous Republic. *Transactions of the Irkutsk State Plague Control Scientific Institute of Siberia and Far East* **15**, 299-309 (in Russian).

**Fernandes FR, Cruz LD, Linhares AX, von Zuben CJ** (2015) Effect of body size on the abundance of ectoparasitic mites on the wild rodent *Oligoryzomys nigripes*. *Acta Parasitologica* **60**, 515-524.

**Fourie LJ, Dutoit JS, Kok DJ, Horak, IG** (1995) Arthropod parasites of elephant-shrews, with particular reference to ticks. *Mammal Review* **25**, 31-37.

**Frank C** (1977) Über die Bedeutung von Laelaps agilis C.L. Koch 1836 (Mesostigmata: Parasitiformae) für die Übertragung von *Hepatozoon sylvatici* Coles 1914 (Sporozoa: Haemogregarinidae. *Zeitschrift Fur Parasitenkunde* **53**, 307-310.

**French TW** (1982) Ectoparasites of the Southeastern shrew, *Sorex longirostris*, and the masked shrew, *S. cinereus*, in Vigo County, Indiana, USA. *Journal of Medical Entomology* **19**, 628-630.

**Frolova GI** (1990) Gamasid mites – ectoparasites of rodents in Volgograd Oblast]. In Medvedev, SG and Shatrov, AB (eds). *Advantages of Medical Entomology and Acarology in USSR*. Leningrad: USSR Academy of Sciences, pp. 153-155 (in Russian).

**Fuenzalida-Araya K, González-Aguayo F, Moreno L, Landaeta-Aqueveque C, Santodomingo A, Silva-de la Fuente C, González-Acuña D** (2022) New records of *Gigantolaelaps wolffsohni* (Mesostigmata: Laelapidae) in Chile, an ectoparasite of *Oligoryzomys longicaudatus* (Rodentia: Cricetidae): Ecological aspects and relation to body size and sex of their host. *Acarologia* **62**, 965-973.

**Furman DP** (1955) *Steptolaelaps* (Acarina: Laelaptidae) a new genus of mites parasitic on neotropical rodents. *Journal of Parasitology* **41**, 519-525.

**Furman DP** (1971) Observations on some laelapid and macronyssid mites in the Fonseca collection (Acari: Mesostigmata). *Papéis Avulsos de Zoologia* **25**, 69-88.

**Furman DP** (1972) Laelapid mites (Laelapidae: Laelapinae) of Venezuela. *Brigham Young University Science Bulletin, Biological Series* **27**, 1-58.

**Garbuzov MA** Ectoparasites of muskrat (Ondatra zibethica L.) and its contacts with other rodents in Khabarovsk Krai. *Transactions of the Irkutsk State Plague Control Scientific Institute of Siberia and Far East* **17**, 143-146 (in Russian).

**Garrett DA, Allred DM** (1971) Mesostigmatid mites from Turkey, with keys to genera and species. *Journal of Medical Entomology* **8**, 292–298. doi: 10.1093/jmedent/8.3.292

**Garrett LE, Strandtmann, RW** (1967) *Tur breviperitremus* n.sp., a new laelapine mite from South Africa. *Journal of Medical Entomology* **4**, 240-246.

**Gettinger D** (1987) Host associations of *Gigantolaelaps* (Acari: Laelapidae) in the Cerrado Province of Central Brazil. *Journal of Medical Entomology* **24**, 559-565.

**Gettinger D** (1992) Host specificity of *Laelaps* (Acari: Laelapidae) in central Brazil. *Journal of Medical Entomology* **29**, 71-77.

**Gettinger D, Owen, RD** (2000) *Androlaelaps rotundus* Fonseca (Acari: Laelapidae) associated with akodontine rodents in Paraguay: a morphometric examination of a pleioxenous ectoparasite. *Revista Brasileira de Biologia* **60**, 425-434.

**Gettinger D, Gardner SL** (2005) Bolivian ectoparasites: a new species of laelapine mite (Acari: Parasitiformes, Laelapidae) from the rodent *Neacomys spinosus*. *Journal of Parasitology* **91**, 49-52.

**Gettinger D, Gardner, SL** (2017) Ectoparasitic mites of the genus *Gigantolaelaps* (Acari: Mesostigmata: Laelapidae) associated with small mammals of the genus *Nephelomys* (Rodentia: Sigmodontinae), including two new species from Peru. *Acarologia* **57**, 755-763.

**Gettinger D, Martins-Hatano F, Lareschi M, Malcolm JR** (2005) Laelapine mites (Acari: Laelapidae) associated with small mammals from Amazonas, Brazil, including a new species from marsupials. *Journal of Parasitology* **91**, 45-48, 498.

**Gil‐Fernández M, Vargas‐Sandoval M, Delfín‐Alfonso CA, Mendoza E, Godínez‐Gómez O, Jiménez‐Lara NK, MacSwiney MC, Carthey A, Blanco‐García A, Le Roux JJ** (2024) Host sweet host: Rodent communities support similar ectoparasite diversity regardless of anthropogenic disturbance. *Journal of Applied Entomology* **148**, 537-552.

**Gill D, Strandtmann RW** (1977) Ectoparasites of the collared lemming (*Dicrostonyx torquatus*) on Bathurst Island, N.W.T., Canada. *Journal of Medical Entomology* **14**, 101-106.

**Goddard J, Baker GT, Norment, BR** (1986) Ectoparasites of the eastern chipmunk (*Tamias striatus*) from Tishomingo County, Mississippi. *Journal of Wildlife Diseases* **22**, 127-129.

**Gómez-Rodríguez RA, Gutíerrez-Granados G, Montiel-Parra G, Rodríguez-Moreno Á , Sánchez-Cordero V** (2015) Diversity and Coexistence of Ectoparasites in Small Rodents in a Tropical Dry Forest. *Biotropica* **47**, 484–490. doi: 10.1111/btp.12229

**Goncharova AA, Buiakova TG** (1962) Ecology of ticks of the family Haemogamasidae Oudemans, 1920 (Parasitiformes, Gamasoidea) of Transbaikalia. *Voprosy ecologii* **8**, 27-29 (in Russain).

**Goncharova AA, Bondarchuk AS, Vershinina ON** (1991) *Gamasid mites – ectoparasites of mammals in Transbaikalia*. Chita: Chita State Medical Institute. (In Russian).

**González-Aguayo F, Fuenzalida-Araya K, Landaeta-Aqueveque C, Moreno Salas L, Santodomingo A, Silva-de la Fuente MC** (2023) Evaluation of the influence of biotic and abiotic factors on the prevalence and abundance of infestations of *Mysolaelaps microspinosus* (Fonseca, 1936) (Mesostigmata: Laelapidae) on *Oligoryzomys longicaudatus* (Bennett, 1832) in Chile. *Acarologia* **63**, 735-743.

**Grokhovskaya IM, Huan-Hoe N** (1961) Gamasid-mites of north Viet-Nam, Part 2. *Zoologicheskii Zhurnal* **40**, 1633–1646. (In Russian).

**Guerra AS, Eckerlin RP, Dowling AP, Durden LA, Robbins RG, Dittmar K, Helgen KM, Agwanda B, Allan BF, Hedlund T, Young HS** (2016) Host-parasite associations in small mammal communities in semiarid savanna ecosystems of East Africa. *Journal of Medical Entomology* **53**, 851-860.

**Guo XG** (1998) Host specificity and host-selection of gamasid mites (Acari: Gamasina). *Systematic and Applied Acarology* **3**, 29–34.

**Guo XG, Speakman JR, Dong WG, Men XY, Qian TJ, Wu D, Qin F, Song WY** (2013) Ectoparasitic insects and mites on Yunnan red-backed voles (*Eothenomys miletus*) from a localized area in southwest China. *Parasitology Research* 112, 3543–3549. https://doi.org/10.1007/s00436-013-3537-6

**Guzmán-Cornejo C, Herrera-Mares A, Ugalde-Medina A, López-Pérez AM, Del Castillo-Martínez L, Acosta-Gutiérrez R, Cabrera-Garrido M, Morales-Malacar JB** (2020) Arthropods Associated with Mammals. Their Importance as Part of the Richness in a Biosphere Reserve in Mexico. *Journal of Medical Entomology* **57**, 780–787. doi: 10.1093/jme/tjz237

**Gvozdev EV, Doszhanov TN, Senotrusova VN, Ushakova GV, eds** (1982) *Parasitic Mites and Insects of Kazakhstan*. Alma-Ata: Nauka Publishers. (In Russian)

**Hadi JR, Stafford EE, Brown RJ, Dennis, DT** (1976) Small mammal ectoparasites from Ancol, Jakarta, Indonesia. *Southeast Asian Journal of Tropical Medicine and Public Health* **7**, 487-489.

**Haitlinger R** (1984) Zgrupowania stawonogow wysrepujace na Sorex araneus L. i Sorex minutus L. w srodowisku lesnym I bezlesnym wzgorz niemczanskich. *Wiadomosci parazytologiczne* **30**, 345-367.

**Haitlinger R** (1988) Haemogamasidae Oudemans, 1926 (Acari, Mesostigmata) Polski. *Polske Pismo Entomologiczne* **58**, 636–661.

**Halliday B** (2024) A collection of mites in the family Laelapidae from rodents in Western Australia. *Records of the Western Australian Museum* 9-12.

**Hamidi K, Bueno-Marí R** (2021) Host-ectoparasite associations; the role of host traits, season and habitat on parasitism interactions of the rodents of Northeastern Iran. *Journal of Asia-Pacific Entomology* **24**, 308-319.

**Harrison A, Robb GN, Alagaili AN, Hastriter MW, Apanaskevich DA, Ueckermann EA, Bennett NC** (2015) Ectoparasite fauna of rodents collected from two wildlife research centres in Saudi Arabia with discussion on the implications for disease transmission. *Acta Tropica* **147**, 1–5, doi: 10.1016/j.actatropica.2015.03.022

**Herrera-Mares A, Guzman-Cornejo C, Garcia-Prieto L, Rebollo-Hernandez A, Leon-Paniagua L, Del Castillo-Martinez L, Montiel-Parra G, Rios-Sais G** (2022a) Acari (Parasitiformes and Acariformes) from Mexico: Analysis of their geographical and host distribution in Rodentia (Cricetidae). *Journal of Medical Entomology* **59**, 1880-1890.

**Herrera-Mares A, Guzman-Cornejo C, Ulloa-Garcia A, Cordoba-Aguilar A, Silva-de la Fuente MC, Suzan, G** (2022b) Mites, rodents, and pathogens: A global review for a multi-species interaction in disease ecology. *Acta Tropica* **232**, 106509.

**Howell L, Jelden K, Rácz E, Gardner SL, Gettinger D** (2016) Arthropods infesting small mammals (Insectivora and Rodentia) near Cedar Point Biological Station in southwestern Nebraska. *Insecta Mundi* **0478**, 1-16.

**Huang LQ, Guo XG, Wu D, Wang QH** (2009) Community structure and spatial distribution of gamadis mites associated with small mammals in Yunnan, China. *Acta Entomologica Sinica* **52**, 1328–1337.

**Huang LQ, Guo XG, Wu D, Zhou DH** (2010) Distribution and Ecological Niches of Gamasid Mites (Acari: Mesostigmata) on Small Mammals in Southwest China. *Psyche* **2010**, 934508. Doi: 10.1155/2010/934508

**Huang LQ, Guo XG, Speakman JR, Dong WG** (2013) Analysis of gamasid mites (Acari: Mesostigmata) associated with the Asian house rat, *Rattus tanezumi* (Rodentia: Muridae) in Yunnan Province, southwest China. *Parasitology Research* **112**, 1967-1972.

**Igolkin NI** (1978) *The complexes of ectoparasites of small mammals of the southeastern part of West Siberia*. Tomsk: The Tomsk State University Press. (In Russian).

**Islam MM, Farag E, Eltom K, Hassan MM, Bansal D, Schaffner F, Medlock JM, Al-Romaihi H, Mkhize-Kwitshana Z** (2021) Rodent Ectoparasites in the Middle East: A Systematic Review and Meta-Analysis. *Pathogens* **10**, 139. <https://doi.org/10.3390/pathogens10020139>

**Jameson EW** (1965) The genus *Laelaps* (Acarina: Laelapidae) in Taiwan. *Journal of Medical Entomology* **53**, 41-53.

**Kaminskienė E, Radzijevskaja J, Stanko M, Balčiauskas L, Paulauskas A** (2023) Associations between different Laelapidae (Mesostigmata: Dermanyssoidea) mites and small rodents from Lithuania. *Experimental and Applied Acarology* **81**, 149–162. <https://doi.org/10.1007/s10493-020-00493-3>

**Kaminskienė E, Radzijevskaja,J, Griciuvienė L, Stanko M, Snegiriovaitė J, Mardosaitė-Busaitienė D, Paulauskas, A** (2023) Molecular identification and phylogenetic analysis of laelapidae mites (Acari: Mesostigmata). *Animals* **13**, 2185.

**Kaura T, Kaur J, Bisht K, Goel S, Lakshmi P, Grover GS, Mewara A, Biswal M** (2022) Vector and rodent surveillance for Orientia tsutsugamushi in north India. *Journal of Vector Borne Diseases* **59**, 348-355.

**Kirillov AA, Kirillova NY, Ruchin AB** (2022) Parasites, bacteria and viruses of the edible dormouse *Glis glis* (Rodentia: Gliridae) in the Western Palaearctic. *Diversity* **14**, 562.

**Korallo NP** (2009) Parasite-Host Links of Gamasid Mites of the genus *Hirstionyssus* (Acari: Parasitiformes: Gamasina) in the South of the West Siberian Plain. *Sibirskiy Ekologicheskiy Zhurnal* **3**, 359–364. (In Russian).

**Korotkova VS** (1959) To the fauna of gamasid and trombiculid mites of Osh Oblast. *Transactions of the Central Asian Scientific Plague Control Institute* **6**, 295-300 (in Russian).

**Kozlovskaia OL, Khamaganov SA** (1980) Ectoparasites of small mammals of the sands edge of Altan-Els and Borig-Del in Mongolian People’s Republic. In Golubinskii EP (ed). *Problems of the Natural Nidality of Plague, part 1.* Irkutsk, pp. 94-95 (in Russian).

**Krasnov BR, Matthee S, Lareschi M, Korallo-Vinarskaya NP, Vinarski MV** (2010) Co-occurrence of ectoparasites on rodent hosts: Null model analyses of data from three continents. *Oikos* **119**, 120-128.

**Kuo CC, Lee PL, Wang HC** (2020) Molecular detection of Rickettsia species and host associations of *Laelaps* mites (Acari: Laelapidae) in Taiwan. *Experimental and Applied Acarology* **81**, 547-559.

**Lange AB, Hamar M** (1961) Gamasoid mites of rodents and insectivores of People's Republic of Rumania. *Nauchnyje Doklady vysschej shkoly. Biologicheskije nauki* **1**, 21–28. (In Russian).

**Lareschi M, Galliari C** (2014) Multivariate discrimination among cryptic mites of the genus *Androlaelaps* (Acari: Mesostigmata: Laelapidae) parasitic of sympatric akodontine rodents (Cricetidae: Sigmodontinae) in northeastern Argentina: Possible evidence of host switch followed by speciation, with the description of two new species. *Experimental and Applied Acarology* **64**, 479-499.

**Lareschi M, González-Acuña D** (2010) Acari, Laelapidae (ectoparasitic mites), central and southern Chile. *Check List* **6**, 546-548.

**Lareschi M, Krasnov BR** (2010) Determinants of ectoparasite assemblage structure on rodent hosts from South American marshlands: The effect of host species, locality and season. *Medical and Veterinary Entomology* **24**, 284-292.

**Laresch, M, Velazco P** (2013) Laelapinae mites (Acari: Parasitiformes: Laelapidae) parasitic of sigmodontine rodents from Northern Peru, with the description of a new species from *Akodon aerosus* (Rodentia: Cricetidae: Sigmodontinae). *Journal of Parasitology* **99**, 189-193.

**Lareschi M, Autino AG, Diaz M, Barquez, RM** (2003) New host and locality records for mites and fleas associated with wild rodents from northwestern Argentina. *Revista De La Sociedad Entomologica Argentina* **62**. 60-64.

**Lareschi M, Gettinger D, Nava S, Abba AM, Merino ML** (2006) First report of mites and fleas associated with sigmodontine rodents from Corrientes Province, Argentina. *Mastozoologia Neotropical* **13**, 251-254.

**Lareschi M, Gettinger D, Venzal JM, Arzua M, Nieri-Bastos FA, Barros-Battesti DM, Gonzalez, EM** (2006) First report of mites (Gamasida: Laelapidae) parasitic on wild rodents in Uruguay, with new host records. *Neotropical Entomology* **35**, 596-601.

**Lee D, Strandtmann RW** (1967) Two new species of *Gigantolaelaps* (Acarina: Laelaptidae) with a key to the females. *Journal of the Kansas Entomological Society* **40**, 25-32.

**Light JE, Durden LA, OConnor BM** (2020) Checklist of ectoparasites of cricetid and heteromyid rodents in México. *Therya* **11**, 79-136.

**Light JE, Durden LA, OConnor BM, Preisser WC, Acosta R, Eckerlin RP** (2020) Checklist of ectoparasites of cricetid and heteromyid rodents in México. *Therya* **11**, 79–136. doi: 10.12933/therya-20-785

**Linardi PM, Krasnov BR** (2013) Patterns of diversity and abundance of fleas and mites in the Neotropics: Host-related, parasite-related and environment-related factors. *Medical and Veterinary Entomology* **27**, 49-58.

**Linardi PM, Botelho JR, Cunha HC (**1985) Ectoparasitos de roedores da regiäo urbana de Belo Horizonte, MG: II. Oscilacöes dos índices de infestacäo em Rattus norvegicus norvegicus. *Memórias do Instituto Oswaldo Cruz* **80**, 227-232.

**Linardi PM, Botelho JR, Cunha HC, Moreira NS** (1984) Ectoparasitos de roedores da regiao urbana de Belo Horizonte, MG. I. Interacao entre ectoparasitos e hospedeiros. *Memórias do Instituto Oswaldo Cruz* **79**, 239-247.

**Linardi PM, Botelho JR, Ximenez A, Padovani, CR** (1991) Notes on ectoparasites of some small mammals from Santa Catarina State, Brazil. *Journal of Medical Entomology* **28**, 183-185.

**Little AJ, Matthee CA, Ueckermann EA, Horak IG, Hui C, Matthee S** (2024). Host and habitat shape ectoparasite diversity on *Mastomys natalensis* and *Mastomys coucha* (Muridae). *Parasitology* **151**, 769–783. <https://doi.org/10.1017/S0031182024000714>

**Liu P, Li G, Zhao N, Liu Q, Liu X, Song X, Shi X, Lun X, Zhang L, Wang J, Lu L** (2024) Climate heterogeneity, season variation, and sexual dimorphism modulate the association between MHC II diversity and parasite variation in striped hamster. *Integrative Zoology* **19**, 1181-1198.

**López-Pérez AM, Pesapane R, Clifford DL, Backus L, Foley P, Voll A, Silva RB, Foley J** (2022) Host species and environment drivers of ectoparasite community of rodents in a Mojave Desert wetlands. *PLOS One* **17**, e0269160.

**Luo LP, Guo XG, Qian TJ, Wu D, Men XY, Dong WG** (2007) Distribution of gamasid mites on small mammals in Yunnan Province, China. *Insect Science* **14**, 71–78.

**Lutermann H, Archer EK, Ueckermann EA, Junker K, Bennett NC** (2020) Surveys and literature review of parasites among African mole-rats: Proposing hypotheses for the roles of geography, ecology, and host phylogenetic relatedness in parasite sharing. *Journal of Parasitology* **106**, 38-45.

**Madinah A, Abang F, Mariana A, Abdullah MT, Mohd-Azlan J** (2014) Interaction of ectoparasites-small mammals in tropical rainforest of Malaysia. *Community Ecology* **15**, 113-120.

**Madinah A, Fatimah A, Mariana A, Abdullah MT** (2011a) A preliminary field survey of ectoparasites of rodents in urban park, Sarawak, Malaysian Borneo. *Tropical Biomedicine* **42**, 803–813.

**Madinah A, Fatimah A, Mariana A, Abdullah MT** (2011b) Ectoparasites of small mammals in four localities of wildlife reserves in Peninsular Malaysia. *Southeast Asian Journal of Tropical Medicine and Public Health* **42**, 803-813.

**Madinah A, Fatimah A, Mariana A, Abdullah MT** (2013) A preliminary field survey of ectoparasites of rodents in urban park, Sarawak, Malaysian Borneo. *Tropical Biomedicine* **30**, 547-551.

**Mairawita M, Mursyid A, Dahelmi D, Diniyati F, Lidia D, Putri N, Arifa MM, Jefrial, Maulana RM** (2023) Co-occurrence of ectoparasites on wild rodents in Sipora Island, Mentawai, Indonesia with the zoonotic potential review. *Biodiversitas* **24**, 6369-6376.

**Mariana A, Zuraidawati Z, Ho TM, Kulaimi BM, Saleh I, Shukor MN, Shahrul-Anuar MS** (2005) A survey of ectoparasites in Gunung Stong forest reserve, Kelantan, Malaysia. *Southeast Asian Journal of Tropical Medicine and Public Health* **36**, 1125-1131.

**Mariana A, Zuraidawati Z, Ho TM, Kulaimi BM, Saleh I, Shukor MN, Shahrul-Anuar MS** (2008) Ticks (Ixodidae) and other ectoparasites in Ulu Muda Forest Reserve, Kedah, Malaysia. *Southeast Asian Journal of Tropical Medicine and Public Health* **39**, 496-506.

**Martins-Hatano F, Gettinger D, Bergallo HG** (2002) Ecology and host specificity of laelapine mites (Acari: Laelapidae) of small mammals in an Atlantic Forest area of Brazil. *Journal of Parasitology* **88**, 36-40.

**Mašán P, Fenda P** (2010) *A Review of the Laelapid Mites Associated with Terrestrial Mammals in Slovakia, with a Key to the European Species (Acari: Mesostigmata: Dermanyssoidea)*. Bratislava: Institute of Zoology, Slovak Academy of Sciences.

**Matthee S, Ueckermann EA** (2008) Ectoparasites of rodents in southern Africa: a new species of *Androlaelaps* Berlese, 1903 (Acari: Parasitiformes: Laelapidae) from *Rhabdomys pumilio* (Sparrman) (Rodentia: Muridae). *Systematic Parasitology* **70**, 185–190. <https://doi.org/10.1007/s11230-008-9130-1>

**Matthee S. Ueckermann EA** (2009) Ectoparasites of rodents in Southern Africa: two new species of *Laelaps* Koch, 1836 (Acari: Laelapidae) ectoparasitic on *Rhabdomys pumilio* (Sparrman) (Rodentia: Muridae). *Systematic Parasitology* 73, 27–35.

**Matthee S, Horak IG, Beaucournu J, Durden LA, Ueckermann EA, McGeoch MA** (2007) Epifaunistic arthropod parsites of the four-striped mouse, *Rhabdomys pumilio*, in the Western Cape Province, South Africa. *Journal of Parasitology* 93, 47–59.

**Matthee S, Horak IG, van der Mescht L, Ueckermann EA, Radloff FGT** (2010a) Ectoparasite diversity on rodents at de Hoop Nature Reserve, Western Cape Province. *African Zoology* **45**, 213–224.

**Matthee S, McGeoch MA, Krasnov BR** (2010b) Parasite-specific variation and the extent of male-biased parasitism; an example with a South African rodent and ectoparasitic arthropods. *Parasitology* **137**, 651-660.

**McAllister CT, Kinsella JM, Durden LA, Greiman SE, Richardson DJ, Tkach VV** (2021) Parasites of southern short-tailed shrews, *Blarina carolinensis* (Mammalia: Eulipotyphla: Soricidae) from Arkansas and Oklahoma, USA. *Comparative Parasitology* **88**, 22-33.

**Meddour S, Mlik R, Dik B, Hastriter MW, Sekour, M** (2022) Ectoparasites of the common gundi (*Ctenodactylus gundi* Rothmann) from the Aures Region, Algeria. *Annals of Parasitology* **68**, 519-529.

*Memórias do Instituto Oswaldo Cruz* **75**, 47-51.

**Milov SS** (1990) To the ectoparasite fauna of rodents of the Aldan Highlands. In Medvedev, SG and Shatrov, AB (eds). *Advantages of Medical Entomology and Acarology in USSR*] Leningrad: USSR Academy of Sciences, pp. 113-114 (in Russian).

**Mitkova K, Berthova L, Kaluz S, Kazimirova M, Burdova L, Kocianova E** (2015) First detections of *Rickettsia helvetica* and *R. monacensis* in ectoparasitic mites (Laelapidae and Trombiculidae) infesting rodents in south-western Slovakia. *Parasitology Research* **114**, 2465-2472.

**Mohd-Taib FS, Asyikha R, Nor SM** (2021) Small mammal assemblages and their ectoparasite prevalence (Acarina) in mangrove forests of Peninsular Malaysia. *Tropical Zoology* **34**, 24-43.

**Moraes GJ, Moreira GF, Freire RAP, Beaulieu F, Klompen H, Halliday B** (2022) Catalogue of the free-living and arthropod-associated Laelapidae Canestrini (Acari: Mesostigmata), with revised generic concepts and a key to genera. *Zootaxa* **5184**, 1–509. <https://doi.org/10.11646/zootaxa.5184.1.1>

**Morlan HB, Strandtmann RW** (1949) The occurrence of neotropical mites in the United States. *Journal of Parasitology* **35**, 217.

**Morozova IV, Bibikova VA, Kalutenova ZP** (1963) On the fauna of gamasid mites of the Sary-Ishikotau Sands. *Zoologicheskiy Zhurnal* **42**, 1872–1876. (In Russian).

**Nadchatram M, Domrow R, Ng CK** (1966) Parasitic Acarina of the mammals. *Bulletin of the National Museum (Singapore)* **34**, 129-140.

**Nazarizadeh M, Martinu J, Novakova M, Stanko M, Stefka J** (2022) Phylogeography of the parasitic mite *Laelaps agilis* in Western Palearctic shows lineages lacking host specificity but possessing different demographic histories. *BMC Zoology*, **7**, 15.

**Nelder MP, Reeves WK** (2005) Ectoparasites of road-killed vertebrates in northwestern South Carolina, USA. *Veterinary Parasitology* **129**, 313-322.

**Netusil J, Zakovska A, Horvath R, Dendis M, Janouskovcova E** (2005) Presence of *Borrelia burgdorferi* sensu lato in mites parasitizing small rodents. *Vector Borne Zoonotic Diseases* **5**, 227-232.

**Netusil J, Zakovska A, Vostal K, Norek A, Stanko M** (2013) The occurrence of *Borrelia burgdorferi* sensu lato in certain ectoparasites (Mesostigmata, Siphonaptera) of *Apodemus flavicollis* and *Myodes glareolus* in chosen localities in the Czech Republic. *Acta Parasitologica* **58**, 337-341.

**Nikulina NA** (2004) *A catalogue of gamasid mites of the Northern Eurasia mammals*. St.-Petersburg: Aktsioner i K°. (In Russian).

**Northover AS, Godfrey SS, Lymbery AJ, Wayne AF, Keatley S, Ash A, Badsha D, Egan SL, Barr J, Thompson RCA, Cooper P** (2023) The parasites of free-ranging terrestrial wildlife from Australia’s south-west. *Australian Journal of Zoology* **71**, ZO23048.

**Obiegala A, Arnold L, Pfeffer M, Kiefer M, Kiefer D, Sauter-Louis C, Silaghi C** (2021) Host-parasite interactions of rodent hosts and ectoparasite communities from different habitats in Germany. *Parasites and Vectors* **14**, 112.

**Okulova NM, Maiorova AD, Zemskaia AA, Kudinov,AA** (1990) [lood-sucking ticks and mites of the Komsomolsk State Reserve (Khabarovsk Krai). In Medvedev, SG and Shatrov, AB (eds).[*Advantages of Medical Entomology and Acarology in USSR*. Leningrad: USSR Academy of Sciences, pp. 121-123 (in Russian).

**Paramasvaran S, Sani RA, Hassan L, Krishnasamy M, Jeffery J, Oothuman P, Salleh I, Lim KH, Sumarni MG, Santhana,RL** (2009) Ectoparasite fauna of rodents and shrews from four habitats in Kuala Lumpur and the states of Selangor and Negeri Sembilan, Malaysia and its public health significance. *Tropical Biomedicine* **26**, 303-311.

**Paulraj PS, Renu G, Ranganathan K, Veeramanoharan R, Kumar A** (2022) Ectoparasites diversity on rodents and shrews at scrub typhus endemic Vellore District of Tamil Nadu, India. *Journal of Arthropod-Borne Diseases* **16**, 51-60.

**Peng PY, Guo XG, Song WY, Hou P, Zou YJ, Fan R, He XS** (2015). Communities of gamasid mites on *Eothenomys miletus* in southwest China. *Biologia* **70**, 674–682. https://doi.org/10.1515/biolog-2015-0080

**Peng PY, Guo XG, Jin DC** (2018) A New Species of *Laelaps* Koch (Acari: Laelapidae) Associated with Red Spiny Rat from Yunnan Province, China. *Pakistan Journal of Zoology* **50**, 1279–1283.

**Piryanik GA** (1962) *Gamasid mites of the mouse-like rodents of the forest-steppe of Ukraine*. Kiev: The Kiev University Press. (In Russian).

**Radovsky FJ** (1994) The evolution of parasitism and the distribution of some dermanyssoid mites (Mesostigmata) on vertebrate hosts. In Houck, MA (ed). *Mites.* Boston, MA: Springer, pp. 186-217.

**Reeves WK, Durden LA, Ritzi CM, Beckham KR, Super PE, OConnor, BM** (2007) Ectoparasites and other ectosymbiotic arthropods of vertebrates in the Great Smoky Mountains National Park, USA. *Zootaxa* **1392**, 31-68.

**Reitblat AG, Kalmykova NP, Emelianov PF** (1974) On eating eggs and larvae of fleas by gamasid mites. In Pilipenko, VG (ed).[*Highly Dangerous Infections in the Caucasus, Issue 1.* Stavropol: USSR Ministry of Health, pp. 176-178 (in Russian).

**Reitblat, AG** (1978) Gamasid mites of Dagestan and contiguous areas of Checheno-Ingooshetia and Stavropol Territory. *Problems of Highly Dangerous Infections* **2**, 70-73 (in Russian).

**Ritzi CM, Whitaker JO** (2003) Ectoparasites of small mammals from the Newport Chemical Depot, Vermillion County, Indiana. *Northeastern Naturalist* **10**, 149-158.

**Ritzi CM, Bartels BC, Sparks DW** (2005) Ectoparasites and food habits of Elliot's short-tailed shrew, *Blarina hylophaga*. *Southwestern Naturalist* **50**, 88-93.

**Roberts M** (1991) The parasites of the Polynesian rat within and beyond New Zealand. *International Journal for Parasitology* 21, 777–783. <https://doi.org/10.1016/0020-7519(91)90145-w>

**Sartbaev SK** (1975) *Ectoparasites of Rodents and Lagomorphs of Kirghizia*. Frunze: Ilim (in Rusian).

**Savchenko E, Lareschi, M** (2022) Integrative taxonomy reveals hidden diversity within the concept of a laelapine mite species (Mesostigmata: Laelapidae) associated with sigmodontine rodents (Cricetidae): Description of three new species of *Laelaps* Koch in the Neotropical region. *Systematic and Applied Acarology* **27**, 2426-2457.

**Savchenko E, Melis M, Lareschi M** (2021) Laelapid mites (Mesostigmata) ectoparasites of *Oligoryzomys* (Rodentia: Cricetidae) in north-eastern and central Argentina. *Mastozoologia Neotropical* **28**, e0526.

**Savchenko E, Espinoza-Carniglia M, Lareschi, M** (2025) New insights into the phylogeny of Laelapinae mites (Mesostigmata: Laelapidae) associated with sigmodontine rodents (Cricetidae) from Argentina, with a morphological characterization of two species of *Mysolaelaps* Fonseca, 1936. *Zootaxa* **5584**, 126-136.

**Saxena VK** (1999) Mesostigmatid mite infestations of rodents in diverse biotopes of central and southern India. *Journal of Parasitology* **85**, 147-149.

**Selmi R, Belkahia H, Dhibi M, Abdelaali H, Lahmar S, Ben Said M, Messadi L** (2021) Zoonotic vector-borne bacteria in wild rodents and associated ectoparasites from Tunisia. *Infection Genetics and Evolution* **95**, 105039.

**Senotrusova VN** (1987) *Gamasid mites – parasites of wild animals of Kazakhstan*. Alma-Ata: Nauka Publishers. (In Russian).

**Silva-de la Fuente MC, Moreno Salas L, Casanueva ME, Lareschi M, Gonzalez-Acuna D** (2020) Morphometric variation of *Androlaelaps fahrenholzi* (Mesostigmata: Laelapidae) associated with three Sigmodontinae (Rodentia: Cricetidae) from the north of Chile. *Experimental and Applied Acarology* **81**, 135-148.

**Skvortsova SS** (1971) To the fauna of gamasid ticks of Aktubinsk Oblast. In Aikimbaev MA (ed). Materials of the 7^th^ Scientific Conference of Plague Control Institutions of Central Asia and Kazakhstan. Alma-Ata: USSR Ministry of Health, pp. 412-414 in Russian).

**Smith AT, Krasnov BR, Horak IG, Ueckermann EA, Matthee S** (2023) Ectoparasites associated with the bushveld gerbil (*Gerbilliscus leucogaster*) and the role of the host and habitat in shaping ectoparasite diversity and infestations. *Parasitology* **150**, 792-804.

**Soloviova AV** 1974. Gamasid mites of the city of Batumi. In Pilipenko, VG (ed). [*Highly Dangerous Infections in the Caucasus, Issue 1*]*.* Stavropol: USSR Ministry of Health, pp. 181-183 (in Russian).

**Sponchiado J, Melo GL, Landulfo GA, Jacinavicius FC, Barros-Battesti DM, Caceres NC** (2015) Interaction of ectoparasites (Mesostigmata, Phthiraptera and Siphonaptera) with small mammals in Cerrado fragments, western Brazil. *Experimental and Applied Acarology* **66**, 369-381.

**Sponchiado J, Melo GL, Martins TF, Krawczak FS, Jacinavicius FC, Labruna MB, Barros-Battesti DM, Caceres NC** (2017) Ectoparasites of small-mammals: Determinants of community structure in South American savannah. *Parasitology* **144**, 475-483.

**Stevens L, Stekolnikov AA, Ueckermann EA, Horak IG, Matthee, S** (2022) Diversity and distribution of ectoparasite taxa associated with *Micaelamys namaquensis* (Rodentia: Muridae), an opportunistic commensal rodent species in South Africa. *Parasitology* **149**, 1229-1248.

**Strandtmann RW** (1946) *Atricholaelaps sigmodoni*, a New Species of mite parasitic on the cotton rat, and notes on the genera *Atricholaelaps* and *Ischnolaelaps* (Acarina: Laelaptidae). *Journal of Parasitology* **32**, 164-169.

**Strandtmann RW** (1949) The blood-sucking mites of the genus *Haemolaelaps* (Acarina; Laelaptidae) in the United States. *Journal of Parasitology* **35**, 325-352.

**Strandtmann RW, Eads RB** (1947) A new species of mite, *Ichoronyssus dentipes* (Acarina: Liponyssinae), from the cotton rat. *Journal of Parasitology* **33**, 51-56.

**Strandtmann RV, Mitchell CJ** (1963) The laelaptine mites of the *Echinolaelaps* complex from the southwest Pacific area. *Pacific Insects* **5**, 541–576.

**Strandtmann RW, Wharton GW** (1958) *A Manual of Mesostigmatid Mites Parasitic on Vertebrates.* College Park, Maryland: Institute of Acarology.

**Strandtmann RW, Yunker CE** (1966) The genus *Hirstionyssus* Fonseca in Panama (Acarina: Dermanyssidae). In Wenzel, RL and Tipton, VJ (eds). *Ectoparasites of Panama.* Chicago: Field Museum of Natural History, pp. 105-124.

**Suntsova NI** (1999) *Flea (Siphonaptera) and Gamasid Mite (Gamasina) Faunas of Small Mammals of Vietnam in the Context of the Plague Problem*. PhD Thesis, Moscow State University, Moscow (in Russian).

**Sylvester TL, Hoffman JD, Lyon, EK** (2012) Diet and ectoparasites of the southern short-tailed shrew (*Blarina carolinensis*) in Louisiana. *Western North American Naturalist* **72**, 586-590.

**Tajedin L, Rassi Y, Oshaghi M, Telmadarraiy Z, Akhavan A, Abai M, Arandian M** (2009) Study on Ectoparasites of *Rhombomys opimus*, the Main Reservoir of Zoonotic Cutaneous Leishmaniasis in Endemic Foci in Iran. *Iran Journal of Arthropod Borne Diseases*, **3**, 41–45.

**Tenorio JM** (1984) Catalog of the world *Echinonyssus* (= *Hirstionyssus*) (Acari: Laelapidae). *International Journal of Entomology* **26**, 260–281.

**Tenorio JM** (1985) Systematics of *Echinonyssus*. II. *E. blanchardi* and *E. confucianus* (Acari: Laelapidae). *International Journal of Acarology* 11, 3–9. doi: 10.1080/01647958508683388

**Tenorio JM, Radovsky FJ** (1973) Two new species of *Trichosurolaelaps* (Acarina: Laelapidae, Hirstionyssinae) from New Guinea. *Journal of Medical Entomology* **10**, 147–157. <https://doi.org/10.1093/jmedent/10.2.147>

**Tenorio JM, Radovsky FJ** (1974) The genus *Mesolaelaps* (Laelapidae: Mesolaelapinae, n. subfam.) with descriptions of two new species from New Guinea. *Journal of Medical Entomology* **11**, 211–222. https://doi.org/10.1093/jmedent/11.2.211

**Tenorio JM, Radovsky FJ** (1979) Review of the subfamily Hirstionyssinae, synonymy of *Echinonyssus* Hirst and *Hirstionyssus* Fonseca, and descriptions of four new species of *Echinonyssus* (Acari: Laelapidae). *Journal of Medical Entomology* **16**, 370–412. <https://doi.org/10.1093/jmedent/16.5.370>

**Tenquist JD, Charleston WAG** (2001) A revision of the annotated checklist of ectoparasites of terrestrial mammals in New Zealand. *Journal of The Royal Society of New Zealand* 31, 481–542.

**Theodor O, Costa M** (1967) *Ectoparasites. Part one.* Jerusalem: The Israel Academy of Sciences and Humanities.

**Tikasingh ES** (2012) Laelapid mites (Acari: Laelapidae) collected from small mammals in Trinidad, Trinidad and Tobago. *Living World, Journal of the Trinidad and Tobago Field Naturalists' Club*. 74-76.

**Till WM** (1963) Ethiopian mites of the genus *Androlaelaps* Berlese s. lat. (Acari: Mesostigmata). *Bulletin of the Natural History Museum. Zoology* 10, 1–104.

**Tipton V** (1960) The genus *Laelaps*, with a Review of the Laelaptidae and a New Subfamily Alphalaelaptinae (Acarina: Laelaptidae). *University of California Publications in Entomology* **16**, 233–356.

**Tipton VJ, Altman RM, Kennan CM** (1966) Mites of the subfamily Laelaptinae in Panama (Acarina: Laelaptidae). In Wenzel, RL and Tipton, VJ (eds). *Ectoparasites of Panama.* Chicago: Field Museum of Natural History, pp. 23-82.

**Trejo JC, López-Berrizbeitia MF** (2024) New findings of mites on small mammals in the Yungas Forest of Argentina. *Therya* **15**, 315-321.

**Uchikawa K** (1971) Studies on mesostigmatid mites parasitic on mammals in Japan. I. *Hirstionyssus apodemi* Zuevsky and *H. tatricus* Mrciak new to Japan, redescriptions of adults and descriptions of deutonymphs. *Japan Journal of Sanitary Zoology* **24**, 261–270.

**Uchikawa K** (1975) Studies on mesostigmatid mites parasitic on mammals in Japan. III. On the identity of *Hirstionyssus apodemi* Zuevsky, 1970, with *H. sunci* Wang, 1962, and the descrtption of *Hirstionyssus japonicus* spec. nov. (Mesostigmata: Laelapidae). *Japan Journal of Sanitary Zoology* **26**, 89–92.

**Urdapilleta M, Galliari CA, Navarro-Febre T, Lareschi M** (2022) Effect of host and environment related factors on the distribution of the ectoparasites of the montane grass mouse (Cricetidae: Sigmodontinae) in the Atlantic Forest ecoregion in northeastern Argentina, with emphasis on laelapids (Mesostigmata). *Revista Mexicana De Biodiversidad* **93**, e933894.

**Várfalvyová D, Stanko M, Miklisova D** (2011) Composition and seasonal changes of mesostigmatic mites (Acari) and fleas fauna (Siphonaptera) in the nests of *Mus specilegus* (Mammalia: Rodentia). *Biologia* **66**, 528-534.

**Viggers KL, Lindenmayer DB, Cunningham RB, Donnelly CF** (1998) The effects of parasites on a wild population of the mountain brushtail possum (*Trichosurus caninus*) in south-eastern Australia. *International Journal for Parasitology* **28**, 747-755.

**Vinarski MV, Korallo-Vinarskaya NP** (2016) An annotated catalogue of the gamasid mites associated with small mammals in Asiatic Russia. The family Laelapidae s. str. (Acari: Mesostigmata: Gamasina). *Zootaxa* **4111**, 223-245.

**Vinarski MV, Korallo-Vinarskaya NP** (2017) An annotated catalogue of the gamasid mites associated with small mammals in Asiatic Russia. The family Haemogamasidae (Acari: Mesostigmata: Gamasina). *Zootaxa* **4273**, 1-18.

**Vinarski MV, Korallo-Vinarskaya NP** (2020) An annotated catalogue of the gamasid mites associated with small mammals in Asiatic Russia. The family Hirstionyssidae (Acari: Mesostigmata: Gamasina). *Zootaxa* **4838**, 102-118.

**Weaver HJ** (2007) *Biodiversity of the parasite fauna of the rodent genera Zyzomys Thomas 1909 and Pseudomys Gray 1832 from Northern Australia.* PhD thesis, Central Queensland University, St Lucia, Australia.

**Weaver HJ, Aberton JG** (2004) A survey of ectoparasite species on small mammals during autumn and winter at Anglesea, Victoria. *Proceedings of the Linnean Society of New South Wales* **125**, 205-210.

**Weaver HJ, Smales LR** (2009) The parasite assemblages of *Zyzomys argurus* (Thomas, 1889) (Muridae:Murinae) from northern Australia. *Australian Journal of Zoology* **57**, 429-432.

**Weaver HJ, Smales LR** (2012) Parasite assemblages of Australian species of *Pseudomys* (Rodentia: Muridae: Murinae). *Journal of Parasitology* **98**, 30-35.

**Webster KN, Hill NJ, Burnett L, Deane, EM** (2014) Ectoparasite infestation patterns, haematology and serum biochemistry of urban‐dwelling common brushtail possums. *Wildlife Biology* **20**, 206-216.

**Wenzel RL, Tipton VJ** (eds). (1966) *Ectoparasites of Panama.* Chicago: Field Museum of Natural History.

**Whitaker JO** (1982) *Ectoparasites of Mammals of Indiana*. Indianopolis, Indiana: The Indiana Academy of Science.

short-tailed shrew, *Blarina carolinensis* (Mammalia, Soricidae), from South Carolina. *Brimleyana* **21***,* 97-105.

**Whitaker JO, Hudson H** (2018) Inhabitants of three nests of the thirteen-lined ground squirrel, *Ictidomys* (formerly *Spermophilus*) *tridecemlineatus* (Mitchill), from Indiana. *Proceedings of the Indiana Academy of Science* **127**, 128-130.

**Whitaker JO, Morales-Malacar JB** (2005) Ectoparasites and other associates (ectodytes) of Mexico. In: **Sánchez-Cordero V and Medellín VY** (eds) *Contributiones Mastozoológicas en Homenaje a Bernardo Villa*. Mexico: Instituto de Biología UNAM; Instituto de Ecología UNAM, pp. 535–666.

**Whitaker JO, Wilson N** (1974) Host and Distribution Lists of Mites (Acari), Parasitic and Phoretic, in the Hair of Wild Mammals of North America, North of Mexico. *The American Midland Naturalist* **91**, 1–67.

**Whitaker JO, Walters BL, Castor LK, Ritzi CM, Wilson N** (2007) Host and distribution lists of mites (Acari), parasitic and phoretic, in the hair or on the skin of North American wild mammals north of Mexico: Records since 1974. *Faculty Publications from the Harold W. Manter Laboratory of Parasitology* 1, 1–173.

**Whitaker JO, Hartman GD, Hein, R** (1994) Food and ectoparasites of the southern

**Yang HJ, Yang ZH, Ren TG, Dong, WG** (2023) Description and phylogenetic analysis of the complete mitochondrial genome in *Eulaelaps silvestris* provides new insights into the molecular classification of the family Haemogamasidae. *Parasitology* **150**, 821-830.

**Yin PW, Guo XG, Jin DC, Fan R, Zhao CF, Zhang ZW, Huang XB, Mao KY** (2021) Distribution and host selection of tropical rat mite, *Ornithonyssus bacoti*, in Yunnan Province of Southwest China. *Animals* **11**, 110.

**Yin PW, Guo XG, Jin DC, Song WY, Zhang L, Zhao CF, Fan R, Zhang ZW, Mao KY** (2021) Infestation and seasonal fluctuation of gamasid mites (Parasitiformes: Gamasida) on Indochinese forest rat, *Rattus andamanensis* (Rodentia: Muridae) in Southern Yunnan of China. *Biology* **10**, 1297.

**Yuan B, He G, Dong, W** (2024) The evolutionary characterization of Gamasida based on mitochondrial genes codon usage pattern. *Parasitology Research* **123**, 30.

**Yudin BS, Krivosheev VG, Belyaev VG** (1976). *Small mammals of the northen Far East*. Novosibirsk: Nauka Publishers. (In Russian).

**Yuldasheva AM, Stanyukovich MK, Fedorova, SZ** (2022) Gamasid mites (Acari: Parasitiformes: Gamasina) of rodents of the valley-foothill zone of the Issyk-Kul basin (Northern Tian-Shan), *Entomological Review* **101**, 1461-1470.

**Yunker CE, Radovsky FJ** (1966) The dermanyssid mites of Panama (Acarina: Dermanyssidae). In Wenzel, RL and Tipton, VJ (eds). *Ectoparasites of Panama.* Chicago: Field Museum of Natural History, pp. 105-124.

**Zaikova FI** (1963) Fauna of gamasid mites of rodents in Transbaikal plague focus *Proceedings of the Irkutsk Plague Control Institute* **6**, 126-129 (in Russian).

**Zhou JX, Guo XG, Song WY, Zhao CF, Zhang ZW, Fan R, Chen T, Lv Y, Yin PW, Jin DC** (2022) Preliminary study on species diversity and community characteristics of gamasid mites on small mammals in three parallel rivers area of China. *Animals* **12**, 3217.

**Zumpt F** (ed). (1961) *The Arthropod Parasites of Vertebrates in Africa South of the Sahara (Ethiopian Region). Volume 1 (Chelicerata).* Johannesburg: G.M.Horne Ltd.
